# Steroids and Sesquiterpenes From Cultures of the Fungus *Phellinus igniarius*

**DOI:** 10.1007/s13659-014-0045-z

**Published:** 2014-11-29

**Authors:** Rong-Hua Yin, Zhen-Zhu Zhao, Xu Ji, Ze-Jun Dong, Zheng-Hui Li, Tao Feng, Ji-Kai Liu

**Affiliations:** 1 State Key Laboratory of Phytochemistry and Plant Resources in West China, Kunming Institute of Botany, Chinese Academy of Sciences, Kunming, 650201 China; 2 University of Chinese Academy of Sciences, Beijing, 100049 China

**Keywords:** Pregnene steroids, Sesquiterpenes, *Phellinus igniarius*, Cytotoxicity, Vascular-activities

## Abstract

Two new steroids, 3*α*,17*α*,19,20-tetrahydroxy-4*α*-methylpregn-8-ene (**1**) and 3*α*,12*α*,17*α*,20-tetrahydroxy-4*α*-methylpregn-8-ene (**2**) and three new sesquiterpenoids, 12-hydroxy-*α*-cadinol (**3**), 3*α*,12-dihydroxy-*δ*-cadinol (**4**), and 3*α*,6*α*-dihydroxyspiroax-4-ene (**5**), have been isolated from cultures of the fungus *Phellinus igniarius*. Their structures were characterized based on extensive spectroscopic data. In preliminary in vitro assays, compounds **3** and **4** exhibited the vascular-activities against phenylephrine-induced vasoconstriction with the relaxing rates of 11.0 % and 7.0 % at 3 × 10^−4^ M, respectively.

**Electronic supplementary material:**

The online version of this article (doi:10.1007/s13659-014-0045-z) contains supplementary material, which is available to authorized users.

## Introduction

**Fig. 1:**
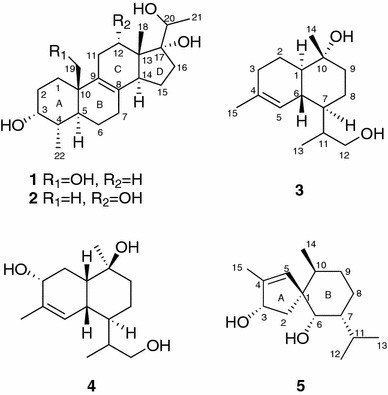
Structures of compounds **1**–**5**

Fungi are biosynthetically talented organisms capable of producing a wide range of chemically diverse and biologically intriguing small molecules. *Phellinus igniarius*, belonging to Polyporaceae family, is widely distributed in Yunnan and Sichuan Provinces of China [1]. It preferably grows on stems of aspen, robur, and birch. Its fruiting body was used to treat fester, abdominalgia, bloody gonorrhea and antidiarrheal in traditional Chinese medicine [2]. Previous chemical investigations on both fruiting bodies and cultures of this fungus reported various secondary metabolites with interesting structures and significant bioactivities [3–7]. Phelligridins D and E showed selective cytotoxicity against a human lung cancer cell line (A 549) and a liver cancer cell line (Bel 7402) [3], while phelligridins H–J, being pyrano[4,3-c] [2] benzopyran-1,6-dione and furo[3,2-c]pyran-4-one derivatives, showed cytotoxic activity against human cancer cell lines and protein tyrosine phosphatase 1B inhibition [4]. A pyrano[4,3-c] [2] benzopyran-1,6-dione derivative and a novel 26-membered macrocyclic metabolite phelligridimer A with antioxidant activities were also isolated from the fruiting bodies [5, 6]. Moreover, several tremulane sesquiterpenes were obtained from the cultures of this fungus, some of which showed significant vascular-relaxing activities against phennylephrine-induced vasoconstriction [7]. To seek for more active molecules, further investigation of this fungus has resulted in the isolation of two pregnene steroids (**1** and **2**) and three sesquiterpenes (**3**–**5**) (Fig. 1). Compounds **1** and **2** are unusual 4-methyl homopregnane derivatives [8], and compound **5** is a rare spiroaxane sesquiterpene which was firstly isolated from the marine sponge *Axinella cannabina* in 1973 [9]. Based on the results of previous biological assays [10, 11], compounds **1**, **2**, and **5** were tested for their cytotoxicity in vitro against five human tumor cell lines, while compounds **3** and **4** were tested for their vascular-activities against phenylephrine-induced vasoconstriction. This paper describes the isolation, structure elucidation and results of biological activities.

## Results and Discussion

**Fig. 2:**
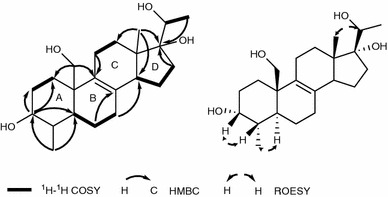
Key^1^H-^1^H COSY, HMBC and ROESY correlations of compound **1**

Compound **1** was isolated as a white amorphous powder. The HREIMS data (*m/z* 364.2611 [M]^+^) indicated the molecular formula C_22_H_36_O_4_, requiring five degrees of unsaturation. The IR absorption bands at 3441 and 1631 cm^−1^ suggested the presence of hydroxy and double bond groups, respectively. The ^1^H NMR spectrum of **1** (Table 1) displayed signals of three methyls (*δ*_H_ 0.74, 0.94, and 1.18). The ^13^C NMR (Table 1) and DEPT spectra of **1** indicated 22 carbon resonances, including an oxygenated methylene carbon (*δ*_C_ 66.3), two oxygenated methines (*δ*_C_ 72.3, 73.1), one oxygenated quaternary carbon (*δ*_C_ 86.4), and a tetrasubstituted double bond (*δ*_C_ 130.9 and 135.1). Apart from one double bond, the remaining four degrees of unsaturation indicated that **1** possessed a four-ring system. Inspection of ^1^H-^1^H COSY correlations resulted in the fragments as shown (Fig. 2). In the HMBC spectrum (Fig. 2), correlations from *δ*_H_ 3.73 (br s, H-3) to *δ*_C_ 26.7 (t, C-1), 30.5 (t, C-2) and 41.8 (d, C-5), *δ*_H_ 0.94 (3H, d, *J* = 6.0 Hz, Me-22) to *δ*_C_ 72.3 (d, C-3), 37.3 (d, C-4) and C-5, *δ*_H_ 3.86 (H, d, *J* = 10.7 Hz, H-19a) and 3.63 (H, d, *J* = 10.7 Hz, H-19) to C-1 and C-5 gave an evidence for a six-member ring A. Besides, correlations of *δ*_H_ 1.69 (H, overlap, H-6a) and *δ*_H_ 2.10 (H, m, H-7a) with *δ*_C_ 130.9 (s, C-8), H-19 with *δ*_C_ 135.1 (s, C-9) and *δ*_C_ 41.7 (s, C-10) indicated the fragments of C-6-C-7-C-8, C-9-C10. Moreover, C-8 and C-9 were connected by the double bond. Hence, another six-member ring B was established. Likewise, in the HMBC spectrum, the correlations from *δ*_H_ 0.74 (3H, s, Me-18) to *δ*_C_ 30.4 (t, C-12) and 48.7 (d, C-14), from H-7 to C-14 and from *δ*_H_ 2.28 (2H, m, H-11) to C-9 indicated the presence of ring C. The last ring D was clearly established by HMBC correlations of Me-18 and *δ*_H_ 1.75 (H, overlap, H-16b) with *δ*_C_ 86.4 (s, C-17). Finally, the backbone of a 6/6/6/5 ring system related to that of 3,17,20-trihydroxy-4-methylpregn-8-en-7-one [7] was deduced. In the ROESY spectrum (Fig. 2), correlations between H-22/H-5 and H-3/H-4 were observed, suggesting that H-3 and H-4 were both *β* oriented. Thus, the structure of compound **1** was elucidated to be 3*α*,17*α*,19,20-tetrahydroxy-4*α*-methylpregn-8-ene, as shown.

**Table 1:** ^1^H and ^13^C NMR data for **1** and **2** (*δ* in ppm, *J* in Hz)

No.	1		2	
	^1^H	^13^C	^1^H	^13^C
1	1.92, m	26.7, CH_2_	1.61, overlap	32.1, CH_2_
	1.50, m		1.48, m	
2	1.52, m	30.5, CH_2_	1.76, m	30.5, CH_2_
	1.80, m		1.71, m	
3	3.73, br s	72.3, CH	3.69, brd (2.5)	72.4, CH
4	1.60, overlap	37.3, CH	1.54, m	36.8, CH
5	1.59, overlap	41.8, CH	1.59, overlap	41.7, CH
6	1.69, overlap	21.7, CH_2_	1.73, m	21.9, CH_2_
	1.35, m		1.30, m	
7	2.10, m	28.1, CH_2_	2.11, overlap	28.4, CH_2_
	2.04, m		2.04, m	
8		130.9, C		129.0, C
9		135.1, C		133.9, C
10		41.7, C		37.5, C
11	2.23, m	25.7, CH_2_	2.33, dt (19.0,4.0)	33.4, CH_2_
			2.09, overlap	
12	1.80, m	30.4, CH_2_	4.17, d (4.0)	73.4, CH
	1.70, overlap			
13		46.7, C		49.9, C
14	2.80,m	48.7, CH	3.23,m	41.6, CH
15	1.74, overlap	24.0, CH_2_	1.83, m	22.9, CH_2_
	1.40, m		1.46, m	
16	2.13, m	38.9, CH_2_	2.07, overlap	38.5, CH_2_
	1.75, overlap		1.79, m	
17		86.4, C		88.6, C
18	0.74, s	14.6, CH_3_	0.63, s	14.4, CH_3_
19	3.86, d (10.7)	66.3, CH_2_	0.96, s	18.5, CH_3_
	3.63, d (10.7)			
20	3.78, q (6.4)	73.1, CH	3.76, q (6.4)	72.6, CH
21	1.18, d (6.4)	18.8, CH_3_	1.29, d (6.4)	18.4, CH_3_
22	0.94, d (6.0)	17.1, CH_3_	0.95, d (6.5)	16.7, CH_3_

Data (*δ*) were measured in methanol-*d*_4_. The assignments were based on DEPT, ^1^H-^1^H COSY, HSQC, and HMBC experiments

Compound **2**, also purified as a white amorphous powder, had the same molecular formula of C_22_H_36_O_4_ as that of compound **1**, according to its HREIMS at *m/z* 364.2605 ([M]^+^). The ^13^C NMR (Table 1) spectroscopic data were similar to those of compound **1**. The main differences were the missing of an oxygenated methylene and the presence of an oxygenated methine in **2**, which revealed the change of substitution of a hydroxy group. It was further confirmed by the HMBC correlations of *δ*_H_ 0.63 (3H, s, Me-18) with the oxygenated methine carbon. The mentioned information suggested that the hydroxy group was located at C-12. On the basis of the ROESY experiment, H-12 was elucidated to be *β* oriented by correlations of H-12 with H-18. Therefore, compound **2** was established to be 3*α*,12*α*,17*α*,20-tetrahydroxy-4*α*-methylpregn-8-ene, as shown.

Compound **3**, a colorless oil, had a molecular formula of C_15_H_26_O_2_ on the basis of HREIMS at *m/z* 238.1931 ([M]^+^). The ^13^C NMR (Table 2) and DEPT spectra of **3** indicated 15 carbon resonances, including three methyls, five methylenes (one oxygenated at *δ*_C_ 67.1), five methines (one *sp*^2^ carbon at *δ*_C_ 123.3), and two quaternary carbon (one oxygenated at *δ*_C_ 72.9 and one *sp*^2^ carbon at *δ*_C_ 136.0). Detailed analysis of NMR data suggested that compound **3** should be a cadinane-type sesquiterpene with a similar structure to that of 15-hydroxy-*α*-cadinol [12]. The main difference between the two compounds was that the hydroxy should be placed at C-12 in **3** rather than at C-15 in 15-hydroxy-*α*-cadinol, which was confirmed by the correlations of *δ*_H_ 2.15 (H, m, H-11) and 0.78 (3H, d, *J* = 7.0 Hz, Me-13) with *δ*_C_ 67.1 (t, C-12) in the HMBC spectrum. Further 2D NMR data supported that the other parts of the structure of **3** were the same as those of 15-hydroxy-*α*-cadinol [12]. Therefore, compound **3** was deduced to be 12-hydroxy-*α*-cadinol.

**Table 2:** ^1^H NMR and ^13^C NMR data for compounds **3–5** (*δ* in ppm, *J* in Hz)

No.	3		4		5	
	^1^H	^13^C	^1^H	^13^C	^1^H	^13^C
1	1.23, overlap	51.0, CH	1.67, dt (14.0, 2.0)	46.3, CH		57.8, C
2	2.06, m	23.9, CH_2_	1.89, dd (11.5, 6.8)	31.8, CH_2_	2.45, dd (13.9, 7.8)	42.8, CH_2_
	1.22, overlap		1.56, overlap		1.56, overlap	
3	1,94 ~ 2.01, m	32.0, CH_2_	4.04, t (8.2)	72.1, CH	4.58, t (6.7)	78.2, CH
4		136.0, C		138.0, C		144.9, C
5	5.50, s	123.3 CH	5.76, d (5.8)	129.6, CH	5.48, s	130.7, CH
6	1.79, m	40.5, C	2.44, m	35.8, CH	3.33, overlap	76.3, CH
7	1.33, m	43.0, CH	1.50, m	43.9, CH	1.03, m	46.9, CH
8	1.49, overlap	23.2, CH_2_	1.43, overlap	23.0, CH_2_	1.65, overlap	25.2, CH_2_
	1.21, overlap		1.43, overlap		1.36, m	
9	1.74, dt (13.0, 3.8)	42.7, CH_2_	1.54, overlap	35.7, CH_2_	1.56, overlap	33.1, CH_2_
	1.47, overlap		1.44, overlap		1.23, m	
10		72.9, C		72.5, C	1.66, overlap	32.6, CH
11	2.15, m	35.4, CH	1.94, m	37.3, CH	1.55, overlap	30.2, CH
12	3,39 ~ 3.46, m	67.1, CH_2_	3.74, dd (10.7, 4.7)	65.2, CH_2_	0.89, d (6.7)	21.5, CH_3_
			3.31, overlap			
13	0.78, d (7.0)	10.6, CH_3_	0.98, d (6.9)	16.3, CH_3_	0.87, d (6.7)	21.1, CH_3_
14	1.07, s	20.5, CH_3_	1.18, s	29.3, CH_3_	0.80, d (6.8)	17.8, CH_3_
15	1.66, s	24.0, CH_3_	1.74, s	19.8, CH_3_	1.75, s	14.0, CH_3_

Data (*δ*) were measured in methanol-*d*_4_. The assignments were based on DEPT, ^1^H-^1^H COSY, HSQC, and HMBC experiments

Compound **4**, a white amorphous powder, possessed the molecular formula C_15_H_26_O_3_, on the basis of its HREIMS at m/z 254.1904 ([M]^+^), 16 mass units higher than that of **3**. The 1D NMR spectroscopic data (Table 2) were quite similar to those of **3**, with the main difference being an oxygenated methane replacing methylene signal confirmed by the HMBC correlations of *δ*_H_ 4.04 (H, t, *J* = 8.2 Hz, H-3) with *δ*_C_ 138.0 (s, C-4) and 129.6 (d, C-5). In the ROESY spectrum, the presence of correlations of H-1/H-3, H-1/H-6 indicated that H-1, H-3 and H-6 were at the same face assigned as *β* orientation. The correlations of H-6 with H-12 suggested that H-7 was at *α* orientation. In addition, the correlations of H-1 with H-9b, Me-14 with H-9a revealed that Me-14 was at *α* orientation. Hence, compound **4** was identified as 3*α*,12-dihydroxy-*δ*-cadinol, as shown.

**Fig. 3:**
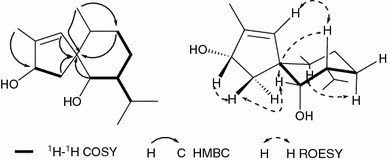
Key^1^H-^1^H COSY, HMBC and ROESY correlations of compound **5**

Compound **5**, a colorless oil, had the molecular formula of C_15_H_26_O_2_ based on its HRESIMS at *m/z* 268.1814 ([M + Na]^+^), which implied the presence of three degrees of unsaturation. The IR spectrum showed absorption bands at 3440 and 1632 cm^−1^, indicating the presence of hydroxy and double bond groups, respectively. The ^13^C NMR (Table 2) and DEPT spectra indicated 15 carbon resonances, classified as four methyls, three methlyenes, six methines (two oxygenated at *δ*_C_ 76.3 and 78.2; one *sp*^2^ carbon at *δ*_C_ 130.7), and two quaternary carbons (one *sp*^2^ carbon at *δ*_C_ 144.9; one *sp*^3^ quaternary carbon at *δ*_C_ 57.8). In consideration of one degree of unsaturation occupied by one double bond, compound **5** was revealed to possess a two-ring system. Analysis of the ^1^H-^1^H COSY spectrum resulted in the deduction of fragments of C-2-C-3, C-7-C-11 and C-6-C-7-C-8-C-9 as shown (Fig. 3). In the HMBC spectrum (Fig. 3), correlations from *δ*_H_ 0.87 (3H, d, *J* = 6.7 Hz, Me-13) to *δ*_C_ 78.2 (d, C-3), *δ*_H_ 2.45 (H, dd, *J* = 13.9, 7.8 Hz, H-2a) and 5.48 (H, s, H-5) to *δ*_C_ 57.8 (s, C-1) supported the foundation of a five-member ring A. Similarly, the other ring B was clearly shown by HMBC correlations of *δ*_H_ 3.33 (H, overlap, H-6), 1.56 (H, overlap, H-9a) and 0.89 (3H, d, *J* = 6.7 Hz, Me-12) with C-1, Me-12 with *δ*_C_ 33.1 (t, C-9). Hence, the two-ring system connected by the spirocarbon C-1 was deduced, which possessed the same skeleton as that of 15-hydroxy-6*α*,12-epoxy-7*β*,10*α*H,11*β*H-spiroax-4-ene [13]. In the ROESY spectrum (Fig. 3), correlations of H-5/H_ax_-7 and H_ax_-7/H_eq_-6 revealed that H-6, H-7 were *β* oriented, while the correlations of H_eq_-6/H-2*β* and H-2*β*/H-3 suggested H-3 was also *β* oriented. However, the presence of correlation of H_ax_-10/H _ax_-8*α* indicated H-10 was *α* oriented. Finally, compound **5** was established as 3*α*,6*α*-dihydroxy-spiroax-4-ene.

Compounds **1**, **2**, and **5** were evaluated for their cytotoxicity against five human cancer cell lines. None was found to possess significant activity with IC_50_ values less than 40 μM. Compounds **3** and **4** were tested for the vascular-activities against phenylephrine-induced vasoconstriction. They exhibited the vascular-activities with the relaxing rates of 11.0 % and 7.0 % at 3 × 10^−4^ M, respectively. It’s worth mentioning that this is the first time to report the vascular-activity of cadinane-type sesquiterpenes.

## Experimental

### General Experimental Procedures

Optical rotations were measured on a Jasco-P-1020 polarimeter. IR spectra were obtained using a Bruker Tensor 27 FT-IR spectrometer with KBr pellets. NMR spectra were acquired with instrument of a Bruker Avance Ш 600 with deuterated solvent signals as internal standards. HREIMS were measured on a Waters Autospec Premier P776 spectrometer. HRESIMS were recorded on an API QSTAR Pulsar spectrometer. Silica gel (200–300 mesh and 80–100 mesh, Qingdao Marine Chemical Inc., China), Sephadex LH-20 and RP-18 gel (20–45 µM, Fuji Silysia Chemical Ltd., Japan) were used for column chromatography (CC). Preparative HPLC was performed on an Agilent 1100 series with a Zorbax SB-C18 (5 μM, 9.4 × 150 mm) column. Fractions were monitored by thin layer chromatography (TLC) (Qingdao Marine Chemical Inc., China) and spots were visualized by heating silica gel plates immersed in H_2_SO_4_ in EtOH, in combination with the Agilent 1200 series HPLC system (Eclipse XDB-C18 column, 5 μM, 4.6 × 150 mm).

### Fungal Material and Cultivation Conditions

Fruiting bodies of *Phellinus igniarius* were collected at Changbai Mountain National Nature Reserve, Antu, Jilin Province, China in 2008 and identified by Prof. Yu-Cheng Dai (Beijing Forestry University). A specimen (No. KIB20081017) was deposited at Kunming Institute of Botany, Chinese Academy of Sciences. The culture medium was composed of glucose (5 %), pork peptone (0.15 %), yeast powder (0.5 %), KH_2_PO_4_ (0.05 %) and MgSO_4_ (0.05 %). The initial PH was adjusted to 6.0 and the fermentation was carried out on a shaker at 150 rpm for 25 days.

### Extraction and Isolation

The cultures (20 L) were filtered through cheesecloth to separate broth and mycelium. The broth was extracted four times with ethyl acetate, while the mycelium was extracted three times with CHCl_3_-MeOH (1:1). The organic layer of both parts were evaporated together to yield a crude extract (9 g). Then this residue was subjected on reverse-phased C18 column eluted with gradient mixture of MeOH and H_2_O (30:70–100:0, v/v). Fractions were collected and monitored by TLC. Similar fractions were pooled to give twelve sub-fractions (A–L). Sub-fraction L was isolated by reverse-phased C18 column eluted with mixture of MeOH and H_2_O (45:55, v/v), then purified by Sephadex LH-20 CC (Me_2_CO) to yield compound **1** (1.4 mg). Sub-fraction G was subjected to Sephadex LH-20 CC (MeOH) and silica gel CC eluted with a petroleum ether-acetone gradient system (6:1, v/v) to give compound **2** (0.8 mg). Sub-fraction I was separated by repeated CC on silica gel and purified by preparative HPLC (MeCN/H_2_O, from 0:100 to 40:60, 10 mL/min, 40 min) to obtain compound **3** (1.8 mg). Sub-fraction A, isolated by Sephadex LH-20 CC (MeOH) and reverse-phased C18 column eluted with mixture of MeOH and H_2_O (30:70, v/v) to yield compound **4** (0.7 mg). Sub-fraction J was subjected to silica gel CC eluted with a petroleum ether-acetone gradient system (4:1, v/v) and Sephadex LH-20 CC (Me_2_CO) to yield compound **5** (1.4 mg).

3*α*,17*α*,19,20-Tetrahydroxy-4*α*-methylpregn-8-ene (**1**): amorphous powder, ${\left[\alpha \right]}_{D}^{21.6}$ −7.2 (*c* 0.13 MeOH); IR (KBr) ν_max_ 3441, 2922, 2852, 1631, 1465, 1384, 1105, 1036 cm^−1^; for ^1^H (600 MHz) and ^13^C NMR (150 MHz) data (methanol-*d*_4_), see Table 1; HREIMS: *m/z* 364.2611 (calcd for C_22_H_36_O_4_, [M]^+^, 364.2614).

3*α*,12*α*,17α,20-Tetrahydroxy-4*α*-methylpregn-8-ene (**2**): amorphous powder, ${\left[\alpha \right]}_{\text{D}}^{21.2}$ −9.7 (*c* 0.07 MeOH); IR (KBr) ν_max_ 3443, 2927, 1634, 1457, 1381, 1065 cm^−1^; for ^1^H (600 MHz) and ^13^C NMR (150 MHz) data (methanol-*d*_4_), see Table 1; HREIMS: *m/z* 364.2605 (calcd for C_22_H_36_O_4_, [M]^+^, 364.2614).

12-Hydroxy-*α*-cadinol (**3**): colorless oil, ${\left[\alpha \right]}_{\text{D}}^{21.8}$ +18.7 (*c* 0.18 MeOH); IR (KBr) ν_max_ 3441, 2926, 1631, 1452, 1382, 1120, 1035 cm^−1^; for ^1^H (600 MHz) and ^13^C NMR (150 MHz) data (methanol-*d*_4_), see Table 2; HREIMS: *m/z* 238.1931 (calcd for C_15_H_26_O_2_, [M]^+^, 238.1933).

3*α*,12-Dihydroxy-*δ*-cadinol (**4**): amorphous powder, ${\left[\alpha \right]}_{\text{D}}^{21.7}$ +9.6 (*c* 0.06 MeOH); IR (KBr) ν_max_ 3424, 2929, 1631, 1436, 1384, 1030 cm^−1^; for ^1^H (600 MHz) and ^13^C NMR (150 MHz) data (methanol-*d*_4_), see Table 2; HREIMS: *m/z* 254.1904 (calcd for C_15_H_26_O_3_, [M]^+^, 254.1882).

3*α*,6*α*-Dihydroxy-spiroax-4-ene (**5**): colorless oil, ${\left[\alpha \right]}_{\text{D}}^{21.1}$ −4.2 (*c* 0.14 MeOH); IR (KBr) ν_max_ 3440, 2926, 2870, 1632, 1459, 1384, 1060 cm^−1^; for ^1^H (600 MHz) and ^13^C NMR (150 MHz) data (methanol-*d*_4_), see Table 2; HRESIMS: *m/z* 268.1814 (calcd for C_15_H_26_O_2_Na, [M + Na]^+^, 268.1831).

### Cytotoxicity Assay

Human myeloid leukemia HL-60, hepatocellular carcinoma SMMC-7721, lung cancer A-549 cells, breast cancer MCF-7 and colon cancer SW480 cell lines were used in the cytoxic assay. All cell lines were cultured in RPMI-1640 or DMEM medium (Hyclone, USA), supplemented with 10 % fetal bovine serum (Hyclone, USA) in 5 % CO_2_ at 37 °C. The cytotoxicity assay was performed according to the MTT (3-(4,5-dimethylthiazol-2-yl)-2,5-diphenyl tetrazolium bromide) method in 96-well microplates [14].

### Vasodilating Activity Assays

Sprague–Dawley rats, weighing 250–350 g, were anaesthetized with pentobarbital sodium (40 mg/kg, i.p.), and the thoracic aorta was removed and placed in Krebs–Henseleit solution (KHS). An aortic ring of about 2–3 mm in length was suspended between two stainless steel hooks in a 5 mL water-jacketed bath containing KHS of the following composition (in mmol/L): NaCl, 120; KCl, 4.7; MgSO_4_·7H_2_O, 1.2; KH_2_PO_4_, 1.2; CaCl_2_·2H_2_O, 2.5; NaHCO_3_, 25; and glucose, 10. The bathing solution was maintained at 37 ± 0.5 °C and was bubbled with 95 % O_2_ and 5 % CO_2_ (pH 7.4) throughout the experiments. One of stainless steel hooks was then connected to a force–displacement transducer (Chengdu instrument factory, Sichuan, China). The initial tension was adjusted to 1.5 g and an equilibration period of 90 min was allowed before commencing the experiments. The resting tension acting in the artery was readjusted periodically until stabilization was achieved. After equilibration, the reactivity of the thoracic aorta was ensured by KCl (60 mmol/L)-induced contraction. When a steady contraction was reached, 10^−5 ^mol/L Acetylcholine (ACh) was added to induce endothelium-dependent relaxation. This step was necessary to verify the integrity of the endothelium.

In order to investigate the effects of various agents on phenylephrine hydrochloride (PE)-induced contraction, when a steady contraction induced by PE (10^−6 ^mol/L) was reached, various agents (3 × 10^−4^ mol/L) was added to the organ bath. The resulting relaxation was expressed as a percentage (%) of the PE-induced steady contraction in the absence of treatment with various agents.

## Electronic supplementary material

Below is the link to the electronic supplementary material.

Supplementary material 1 (DOCX 2983 kb)

## References

[1] X.L. Mao. The Macrofungi in China. Henan Science and Technology Publishing House: Zhengzhou 477 (2000)

[2] Mo SY, Yang YC, Shi JG (2003). China J. Chin. Mater. Med..

[3] Mo SY, Wang S, Zhou G, Yang Y, Li Y, Chen X, Shi JG (2004). J. Nat. Prod..

[4] Wang Y, Shang XY, Wang SJ, Mo SY, Li S, Shi JG, He L (2007). J. Nat. Prod..

[5] Wang Y, Mo SY, Wang SJ, Li S, Yang YC, Shi JG (2005). Org. Lett..

[6] Wang Y, Wang SJ, Mo SY, Li S, Yang YC, Shi JG (2005). Org. Lett..

[7] Wu XL, Lin S, Zhu CG, Yue ZG, Yu Y, Dai JG, Shi JG (2010). J. Nat. Prod..

[8] Habermehl G, Hundrieser HJ (1983). Naturwissenschaften.

[9] Cafieri F, Fattorusso E, Magno S, Santacroce C, Sica D (1973). Tetrahedron.

[10] Ding JH, Feng T, Li ZH, Li L, Liu JK (2012). Nat. Prod. Bioprospect..

[11] Yang XY, Feng T, Wang GQ, Ding JH, Li ZH, Li Y, He SH, Liu JK (2014). Phytochemistry.

[12] Kuo YH, Chen CH, Chien SC, Lin YL (2002). J. Nat. Prod..

[13] Liu DZ, Jia RR, Wang F, Liu JK, Naturforsch Z (2008). B.

[14] Mosmann TJ (1983). Immunol. Methods.

