# Supplementary material for: Steroids and Sesquiterpenes From Cultures of the Fungus Phellinus igniarius
Source: Nat Prod Bioprospect. 2014 Nov 29;5(1):17–22. doi: 10.1007/s13659-014-0045-z (PMC4327997; doi:10.1007/s13659-014-0045-z)

Supporting Information

**Steroids and Sesquiterpenes from cultures of the fungus *Phellinus igniarius***

Rong-Hua Yin^a,b^, Zhen-Zhu Zhao^a,b^, Xu Ji^a^, Ze-Jun Dong^a^, Zheng-Hui Li^a^, Tao Feng^a^, Ji -Kai Liu^a,^*

^a^ *State Key Laboratory of Phytochemistry and Plant Resources in West China, Kunming Institute of Botany, Chinese Academy of Sciences, Kunming 650201, PR China*

*^b^ University of Chinese Academy of Sciences, Beijing 100049, PR China*

**Corresponding author: jkliu@mail.kib.ac.cn*

**Contents**

Figure 1S-7S: NMR and MS spectra of compound **1** ……………………….p2S-5S

Figure 8S-14S: MR and MS spectra of compound **2** ……………………...p6S-9S

Figure 15S-21S: NMR and MS spectra of compound**3** ………………….p10S-13S

Figure 22S-28S: NMR and MS spectra of compound **4**………………….p14S-17S

Figure29S-35S: NMR and MS spectra of compound**5**………………….p18S-21S

Figure 1S. ^1^H NMR (600 MHz, methanol-*d_4_*) spectrum of compound **1**


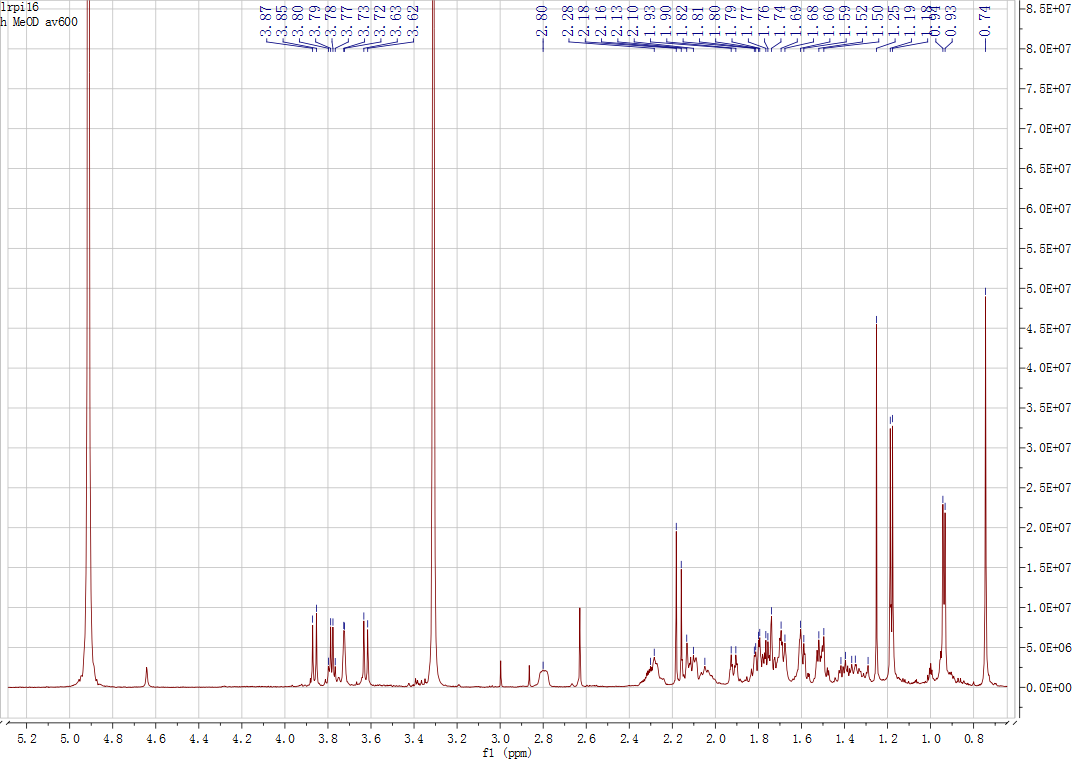


Figure 2S. ^13^C NMR (150 MHz, methanol-*d_4_*) spectrum of compound **1**

**
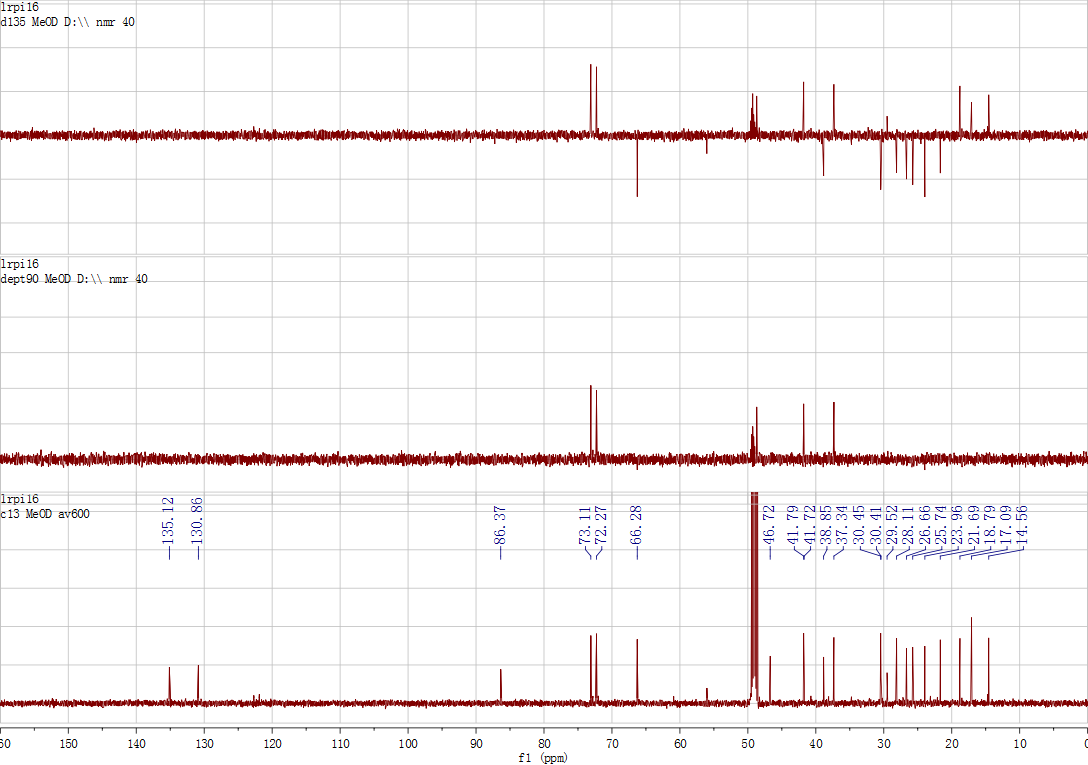
**

Figure 3S. ^1^H-^1^H COSY spectrum of compound **1**


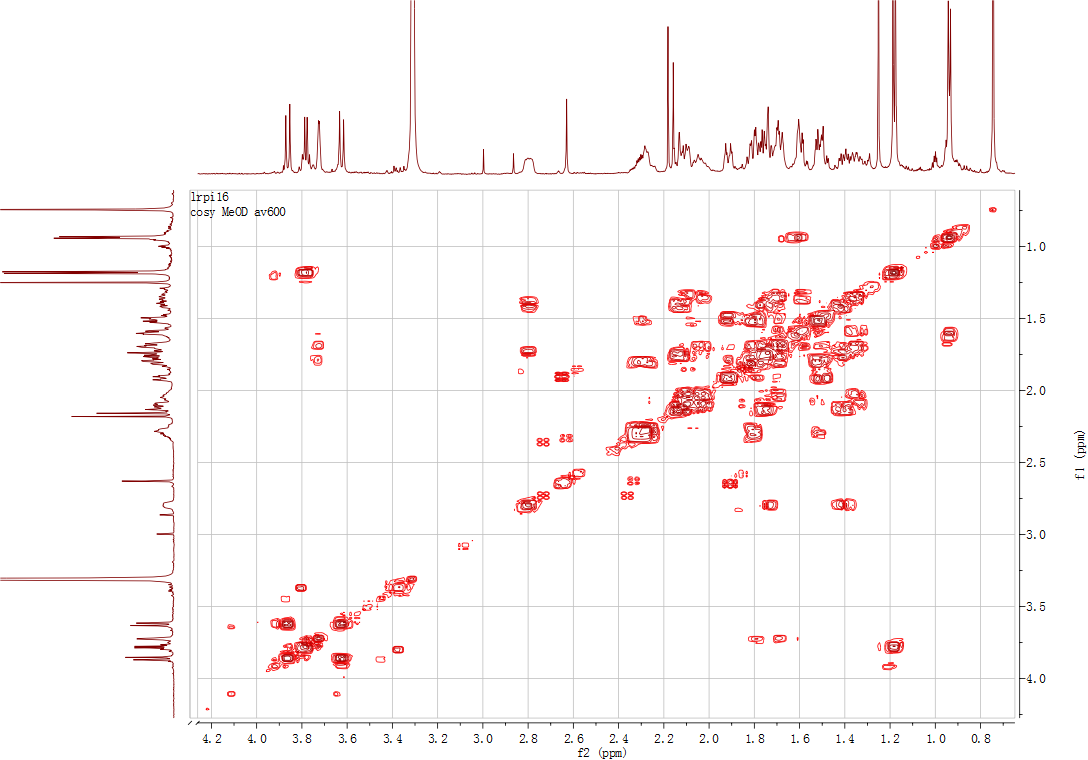


Figure 4S. HQSC spectrum of compound **1**

**
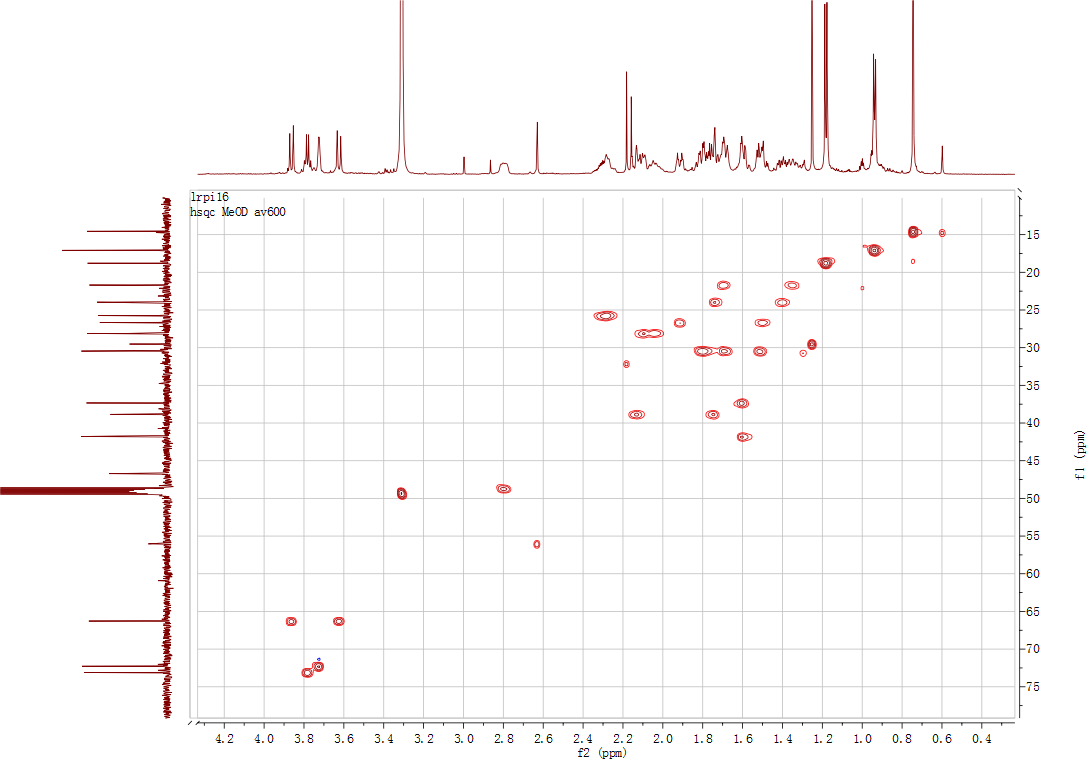
**

Figure 5S. HMBC spectrum of compound **1**

**
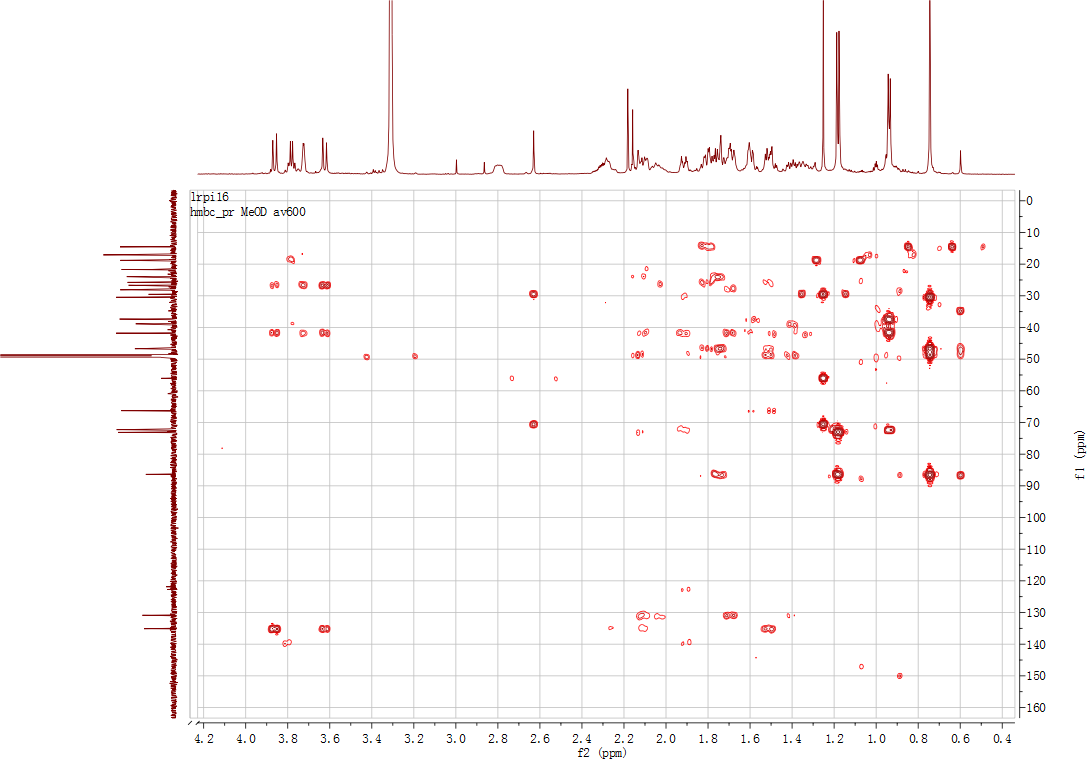
**

Figure 6S. ROESY spectrum of compound **1**

**
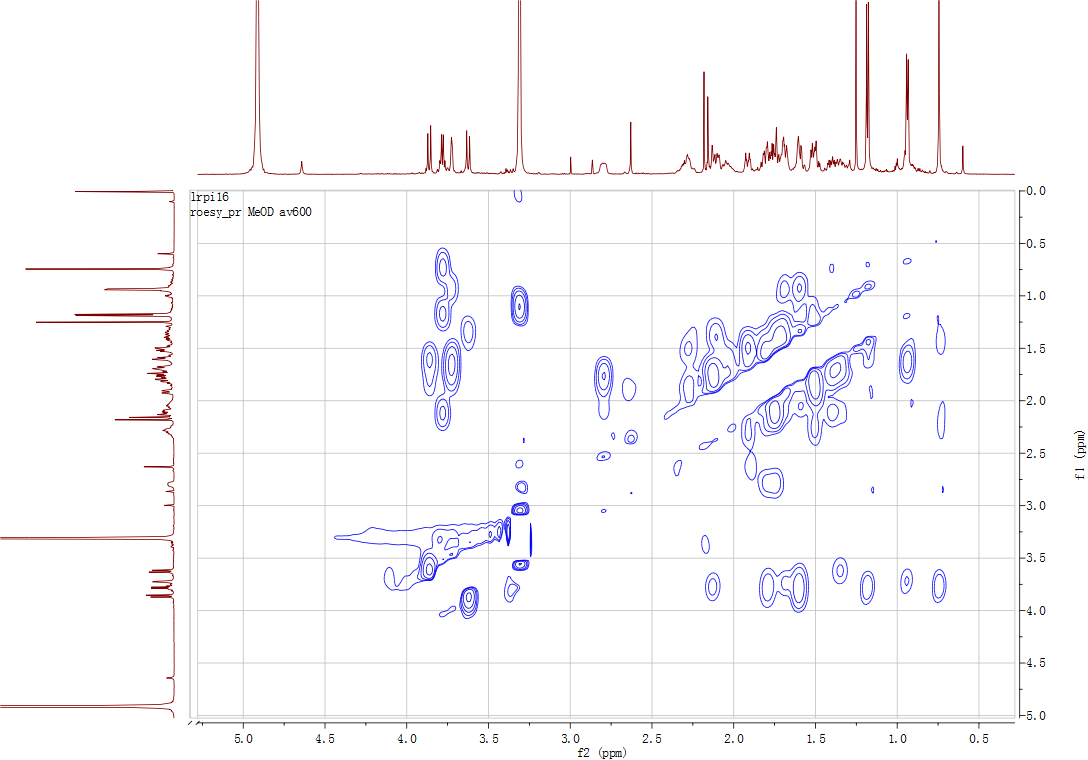
**

Figure 7S. HREIMS of compound **1**

**
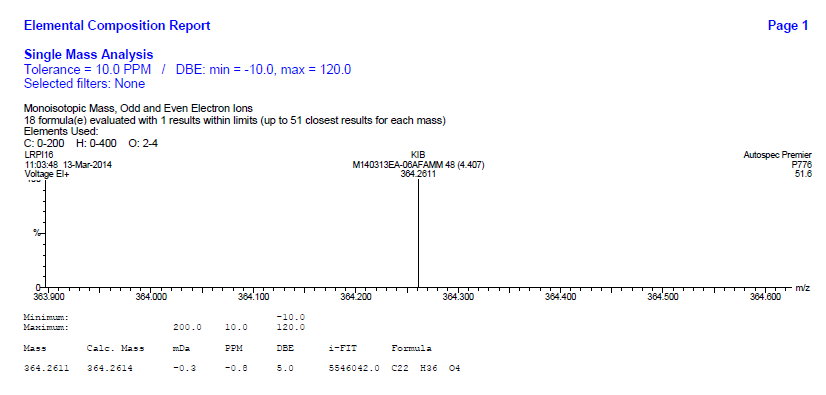
**

Figure 8S. ^1^H NMR (600 MHz, methanol-*d_4_*) spectrum of compound **2**


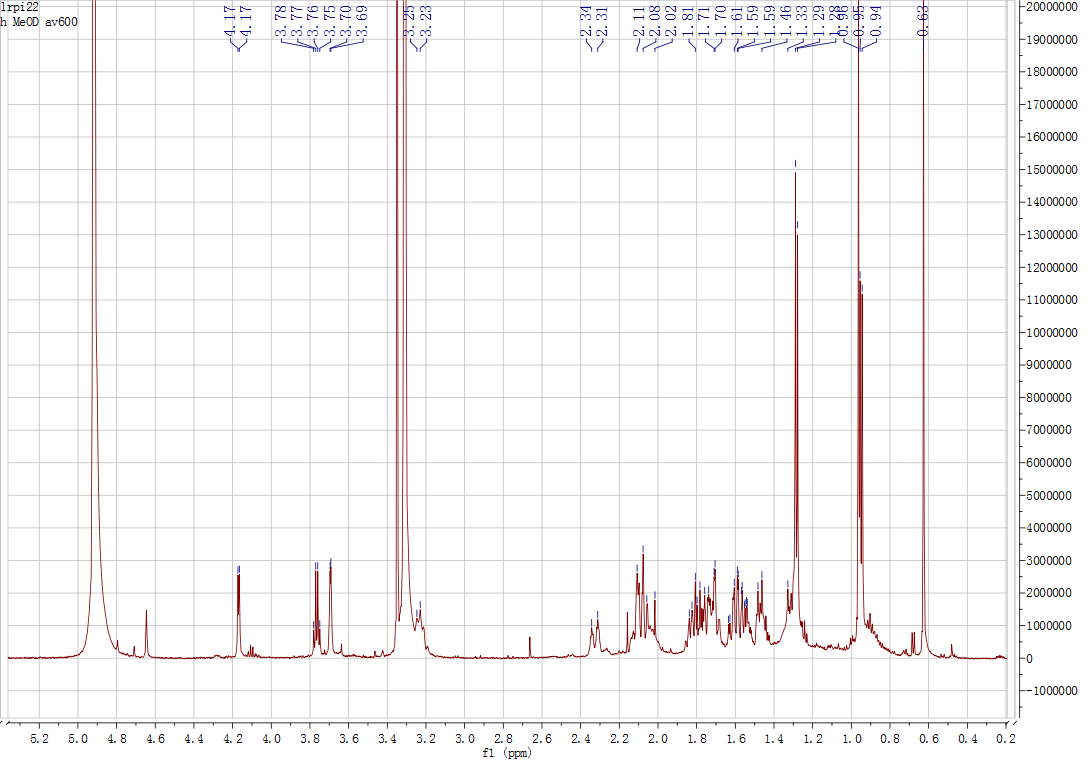


Figure 9S. ^13^C NMR (150 MHz, methanol-*d_4_*) spectrum of compound **2**


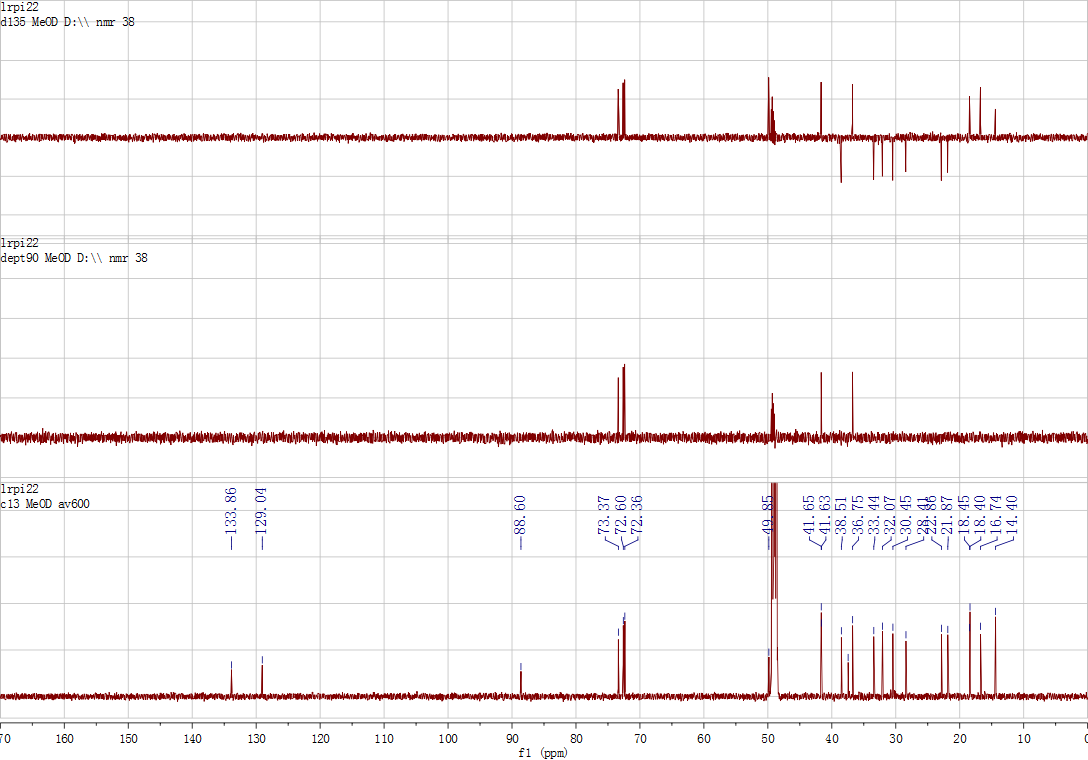


Figure 10S.^1^H-^1^H COSY spectrum of compound **2**


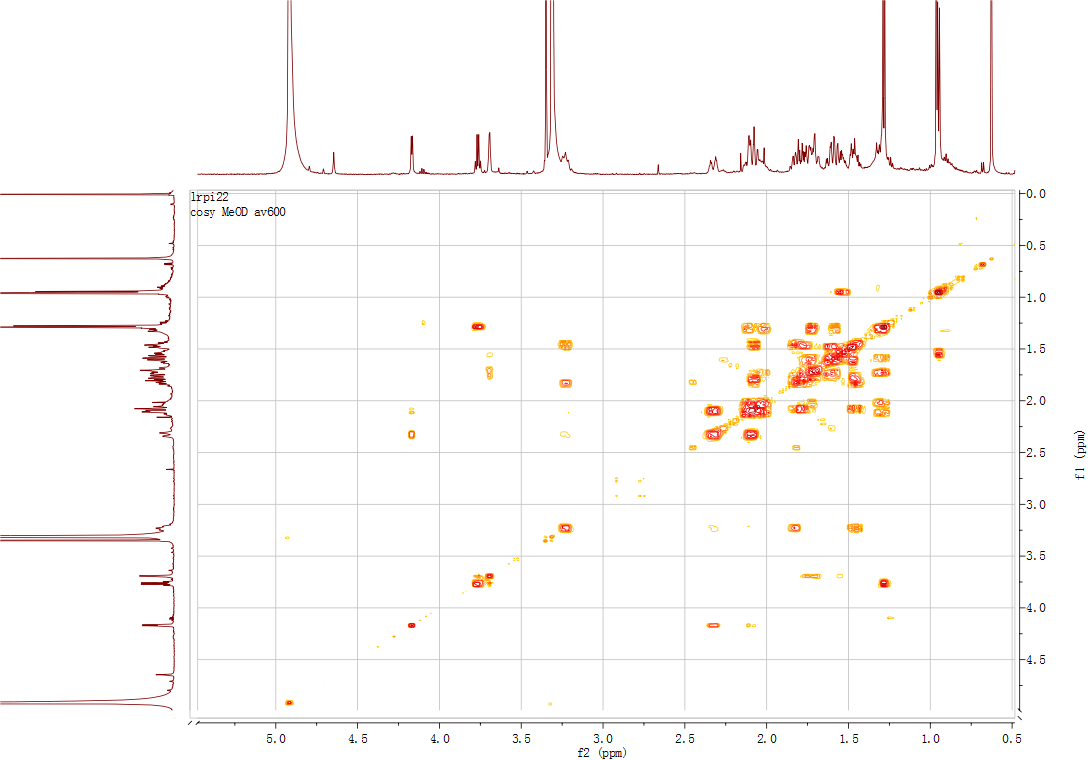


Figure 11S.HSQC spectrum of compound **2**


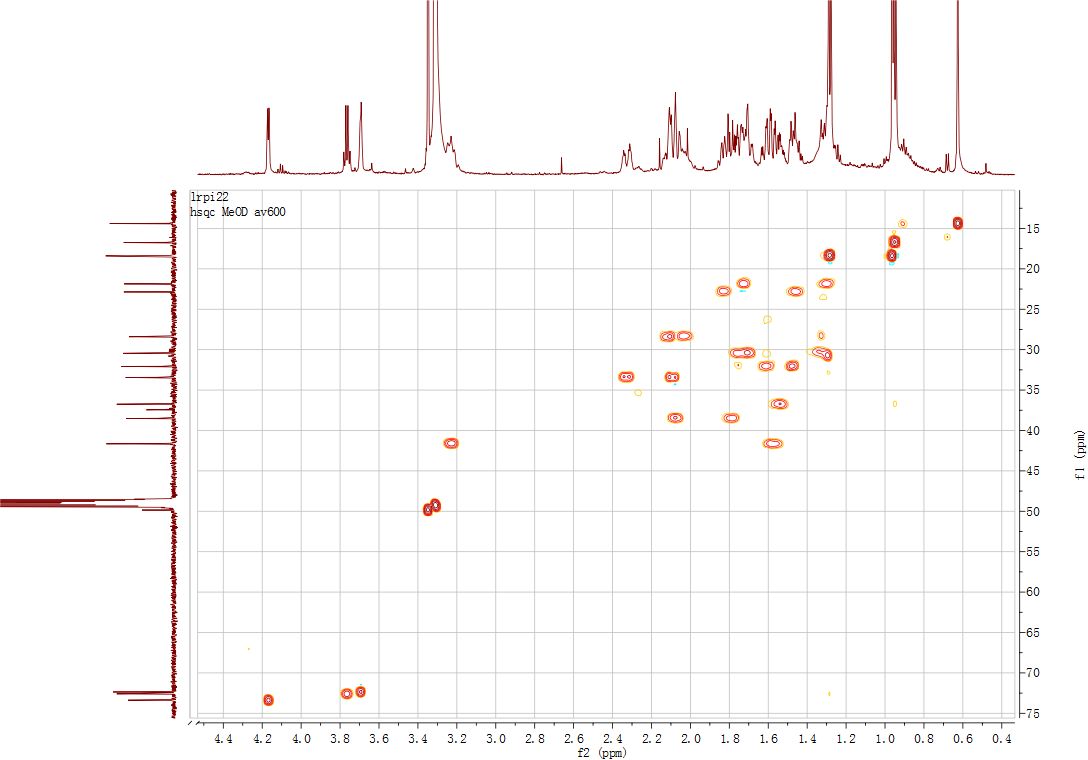


Figure 12S. HMBC spectrum of compound **2**


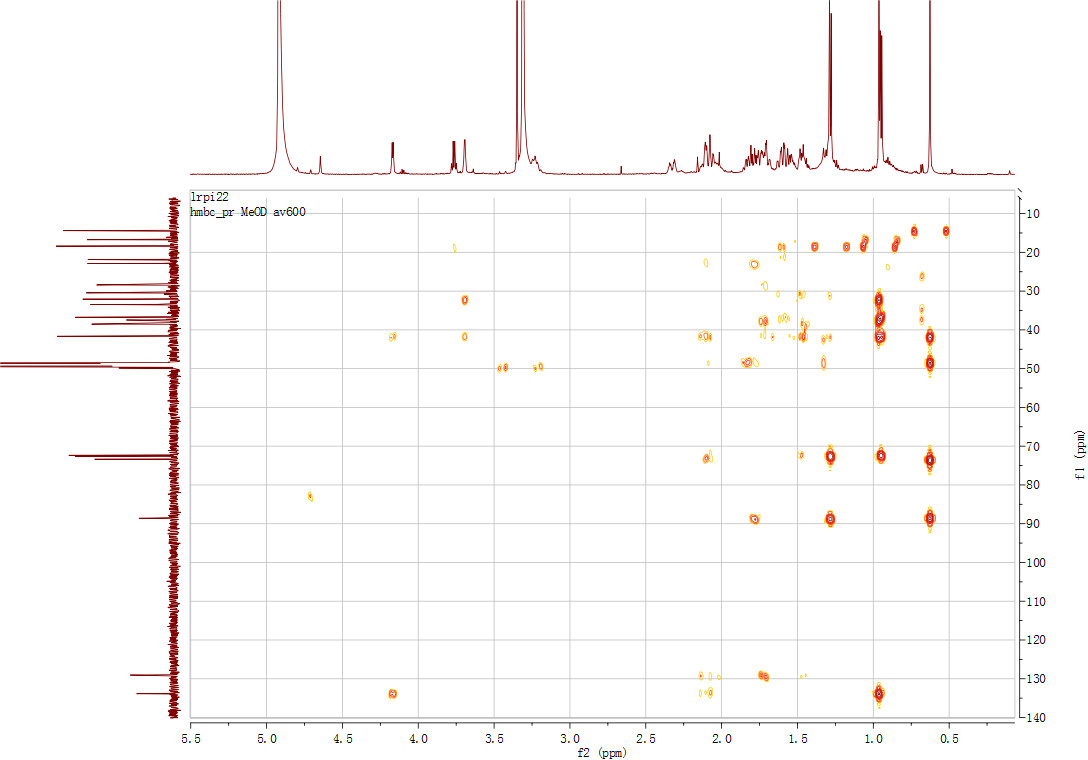


Figure 13S. ROESY spectrum of compound **2**


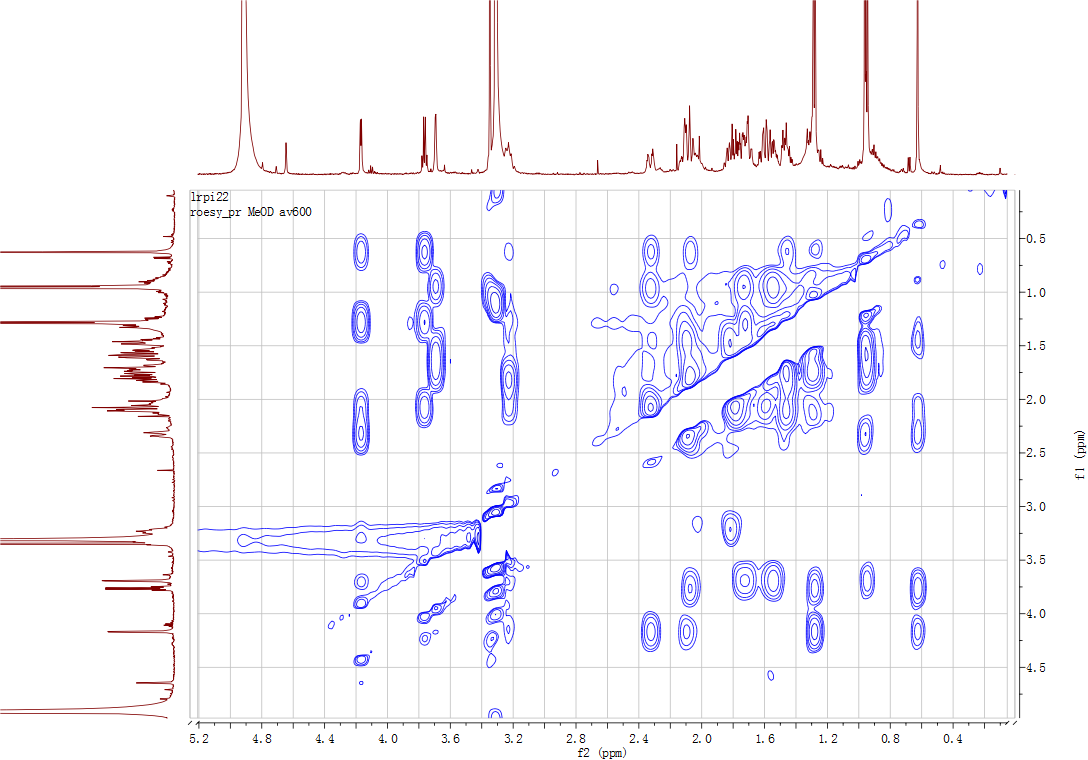


Figure 14S. HREIMS of compound **2**


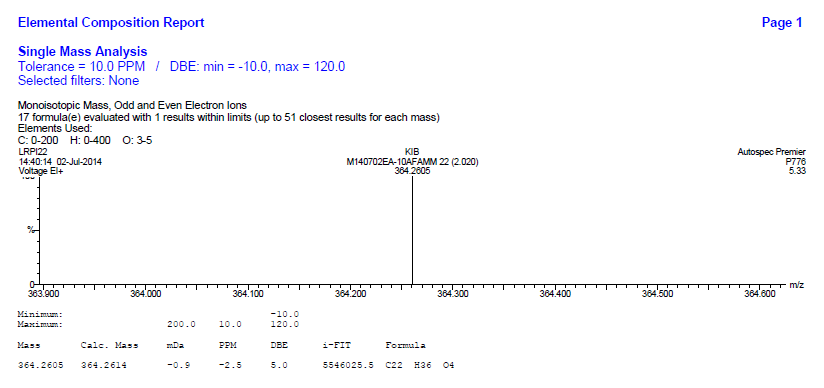


Figure 15S. ^1^H NMR (600 MHz, methanol-*d_4_*) spectrum of compound **3**


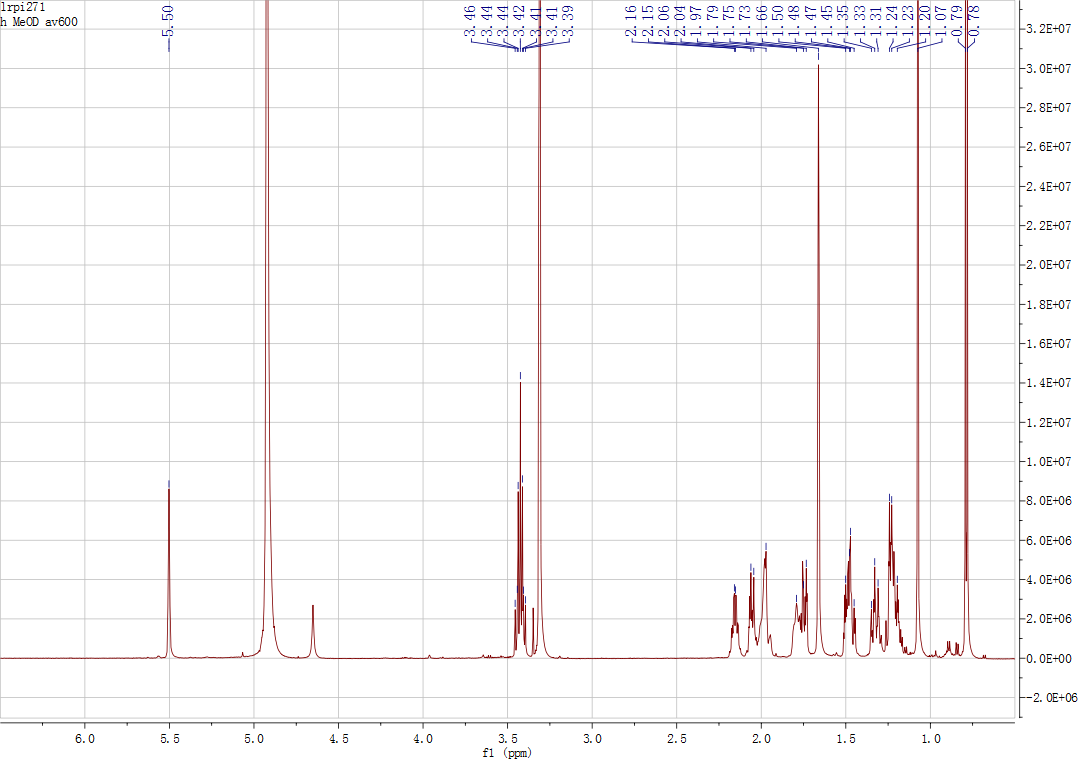


Figure 16S. ^13^C NMR (150 MHz, methanol-*d_4_*) spectrum of compound **3**


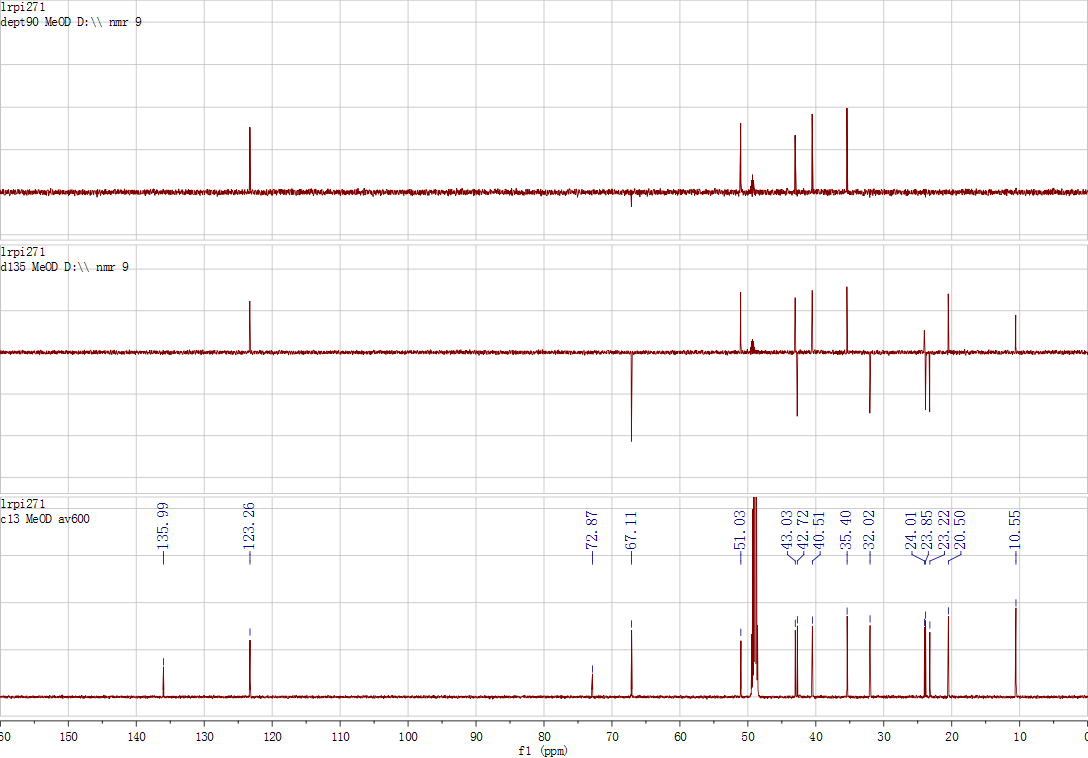


Figure 17S. ^1^H-^1^H COSY spectrum of compound **3**


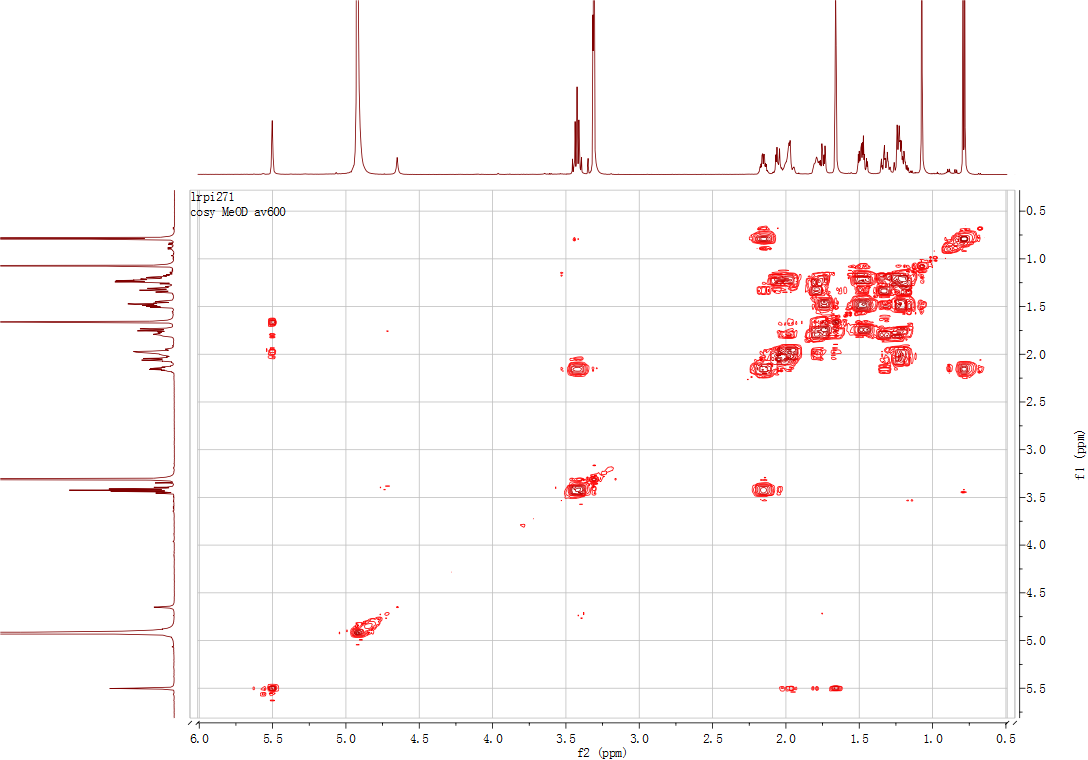


Figure 18S. HQSC spectrum of compound **3**


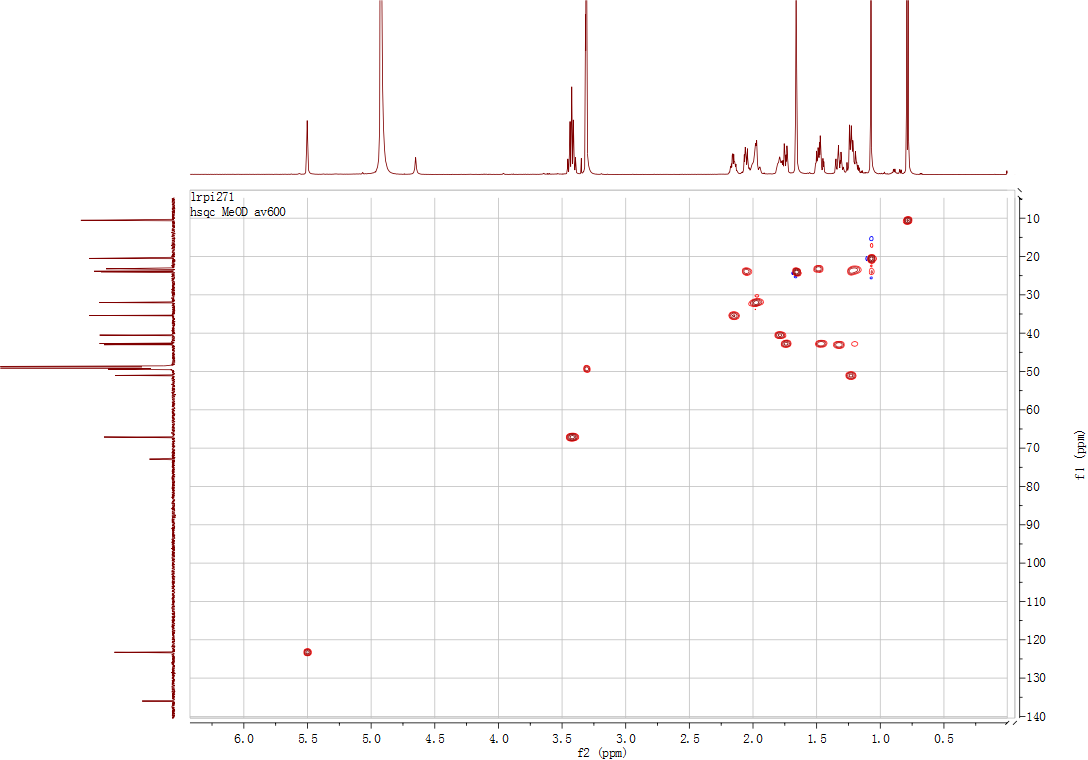


Figure 19S. HMBC spectrum of compound **3**


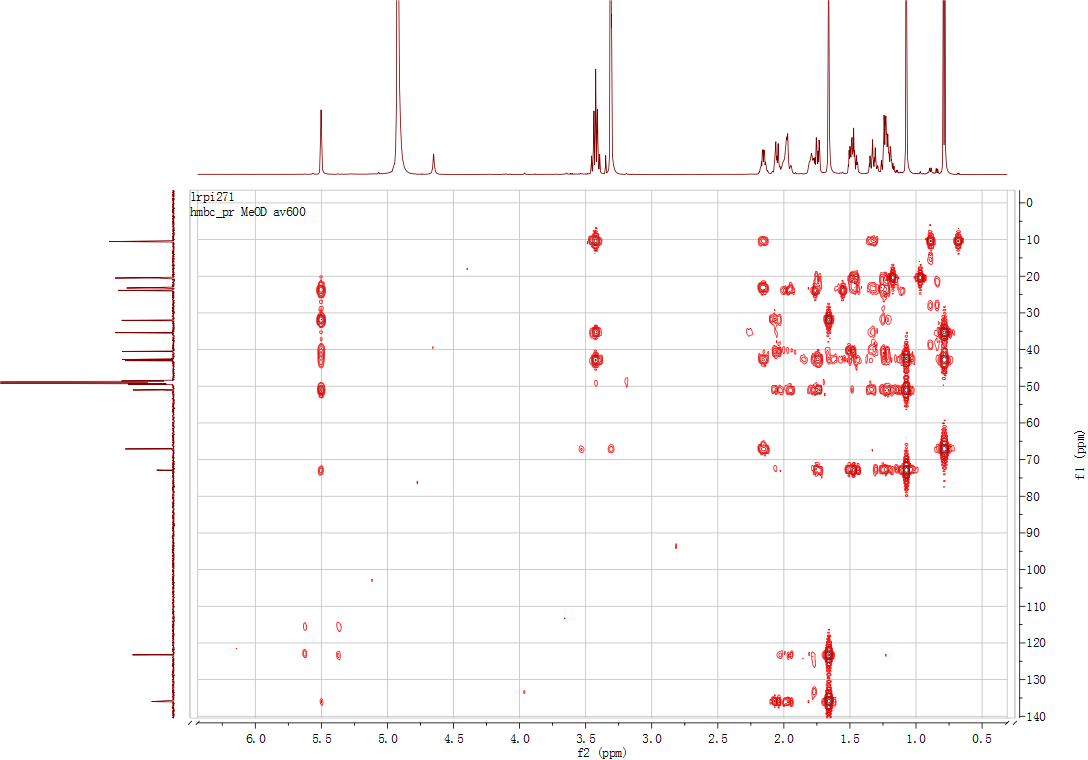


Figure 20S. ROESY spectrum of compound **3**


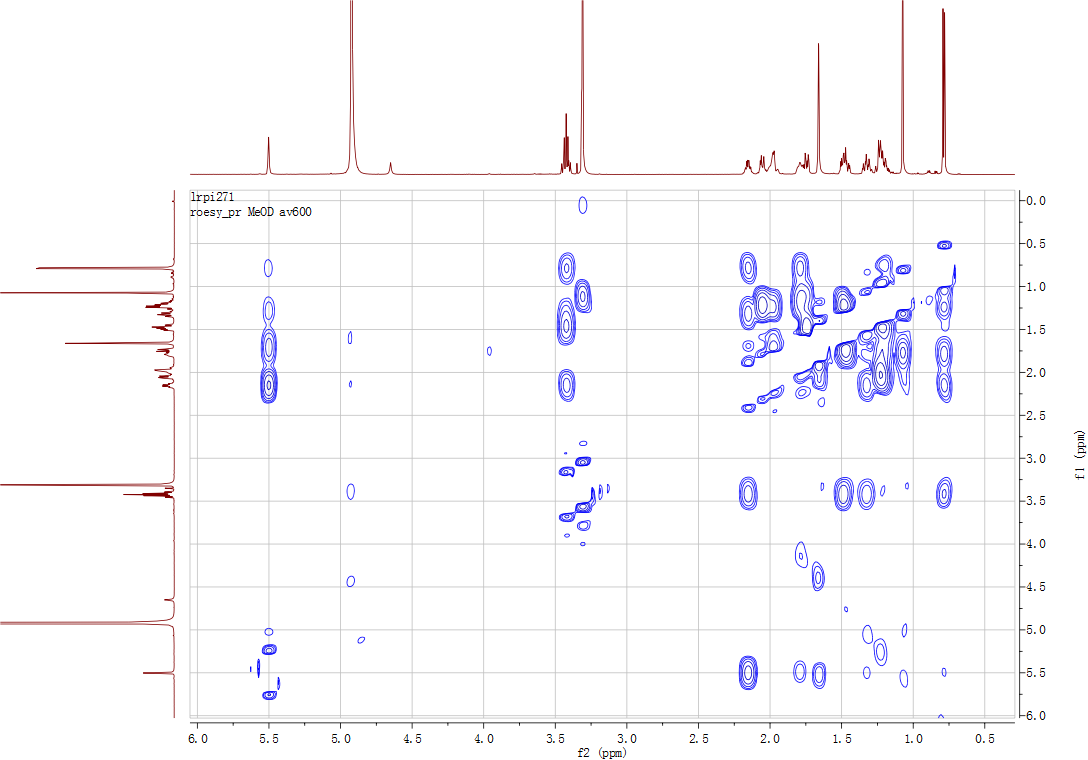


Figure 21S. HREIMS of compound **3**


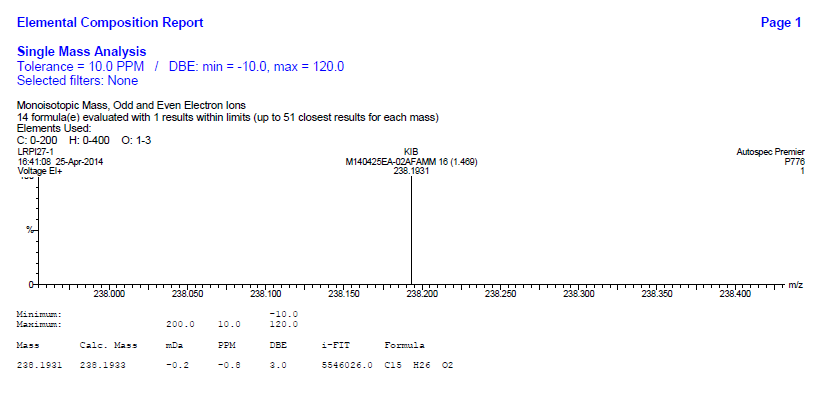


Figure 22S. ^1^H NMR (600 MHz, methanol-*d_4_*) spectrum of compound **4**


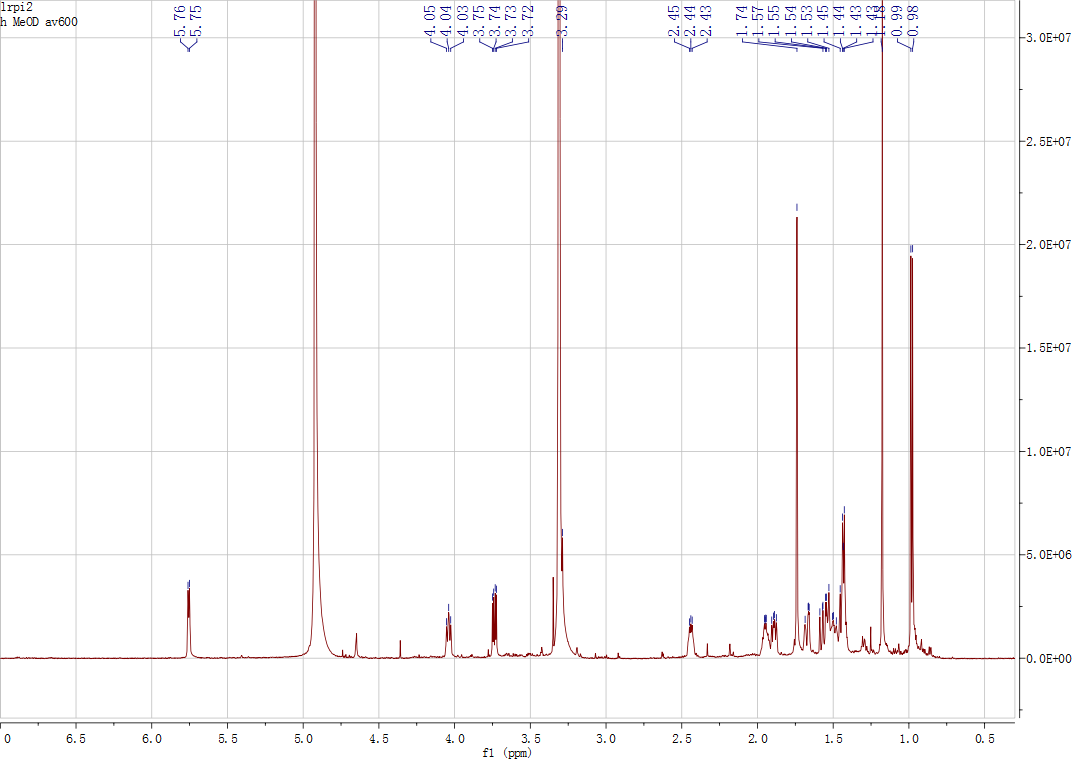


Figure 23S. ^13^C NMR (150 MHz, methanol-*d_4_*) spectrum of compound **4**


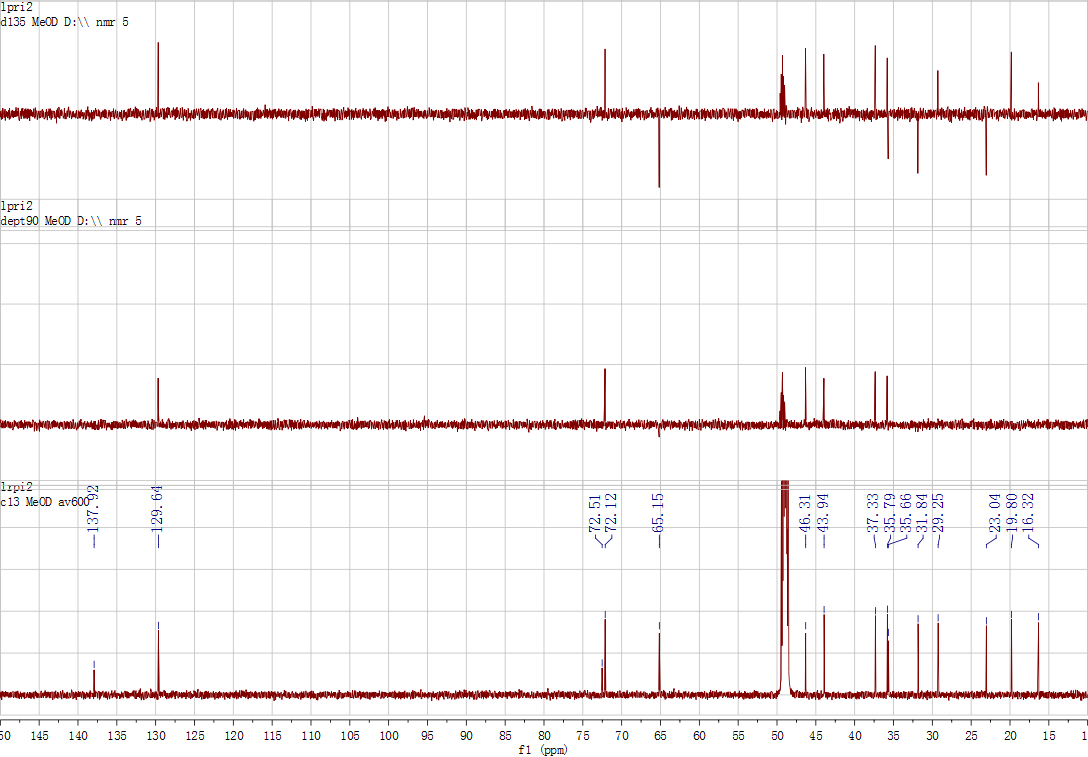


Figure 24S. ^1^H-^1^H COSY spectrum of compound **4**


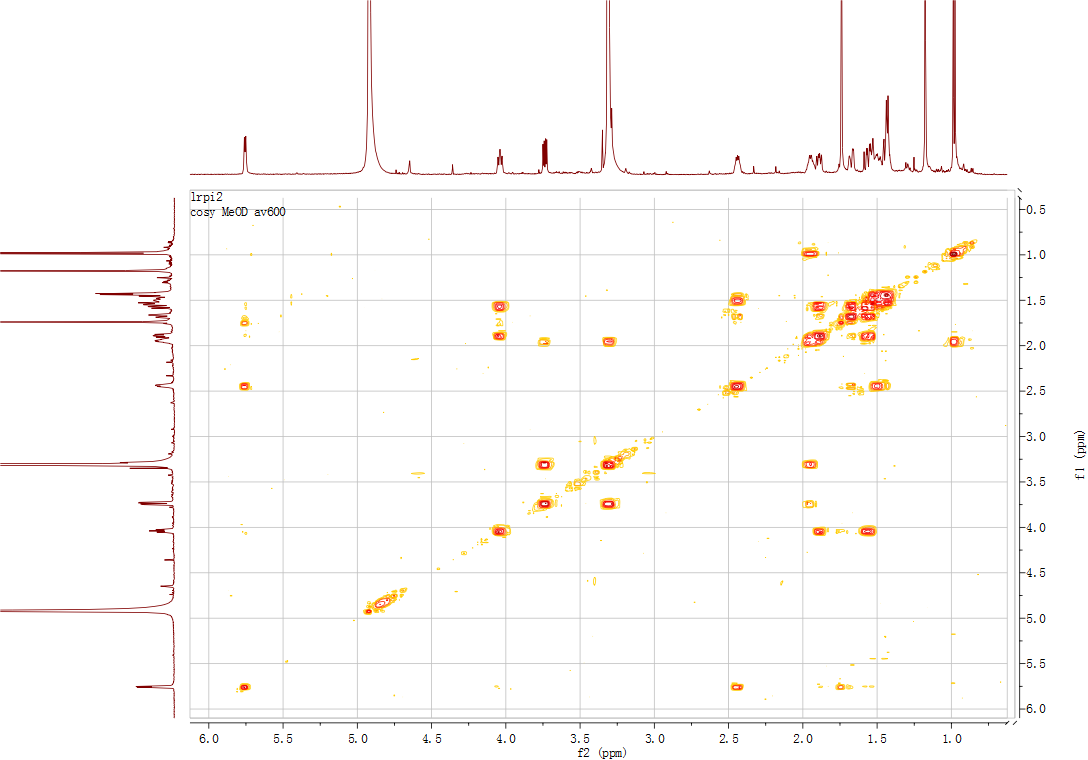


Figure 25S. HQSC spectrum of compound **4**


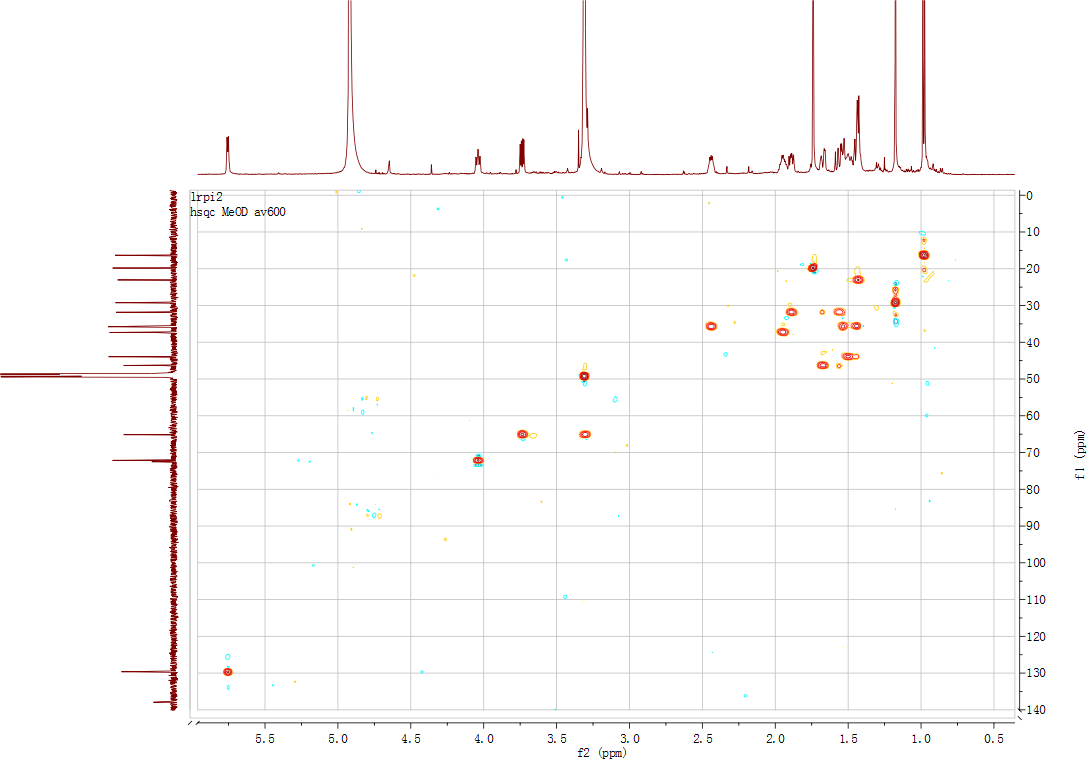


Figure 26S. HMBC spectrum of compound **4**


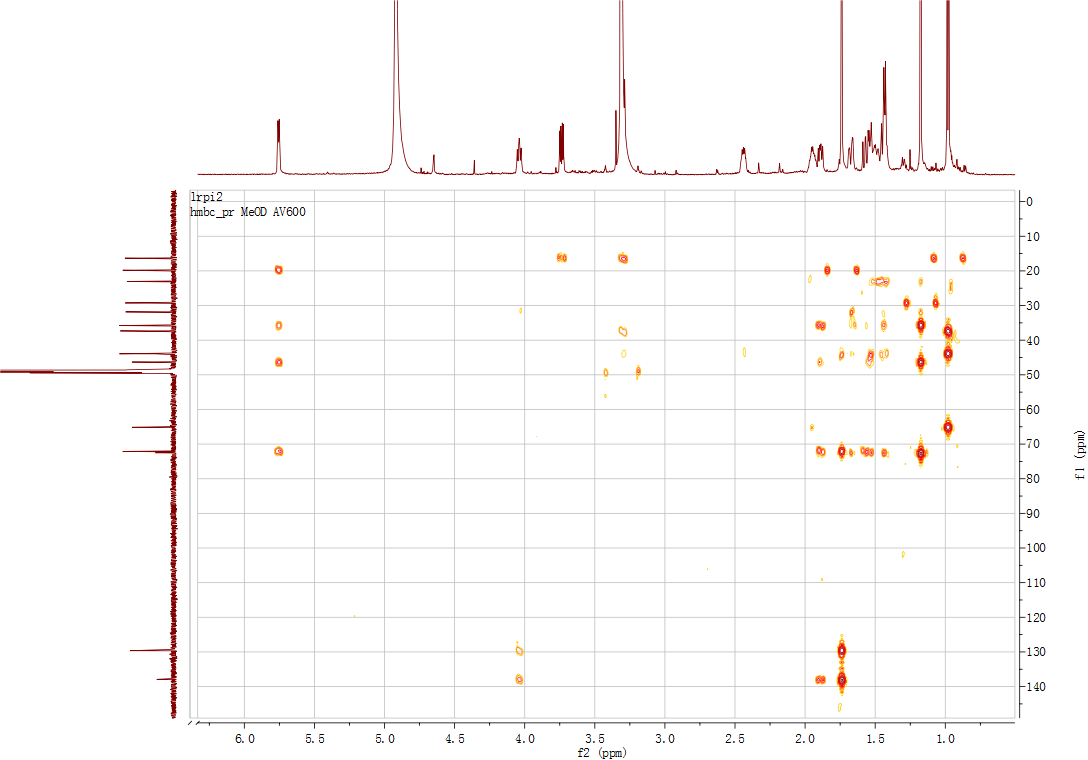


Figure 27S. ROESY spectrum of compound **4**


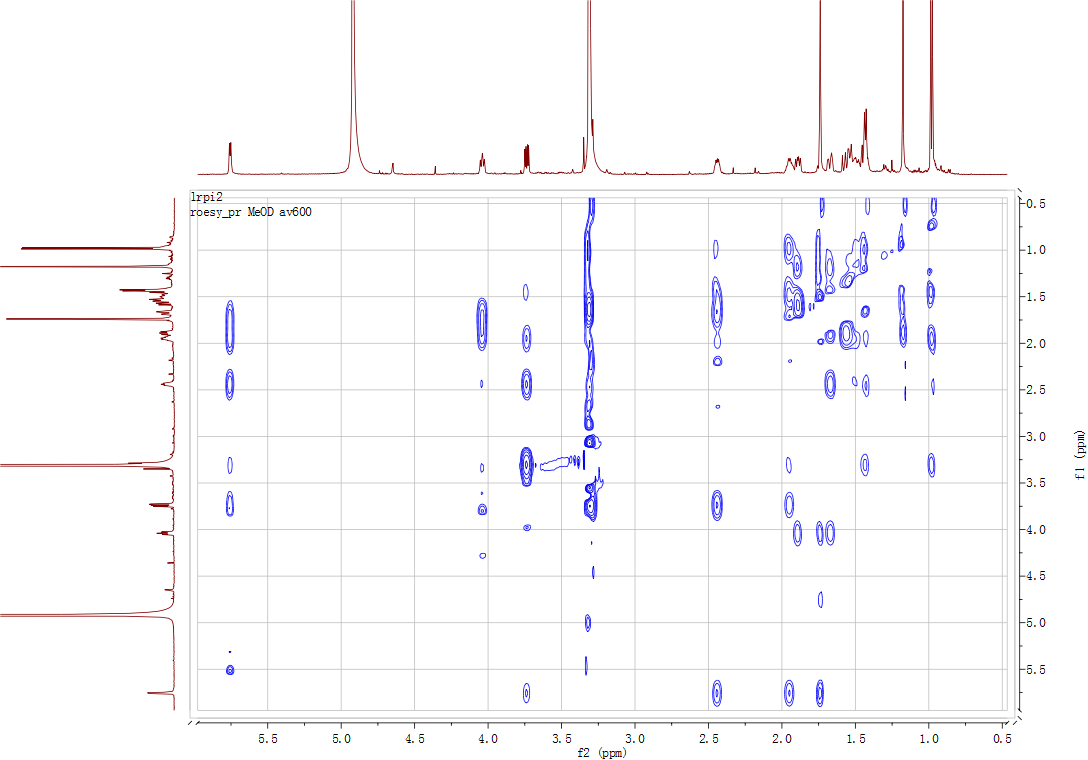


Figure 28S. HREIMS of compound **4**


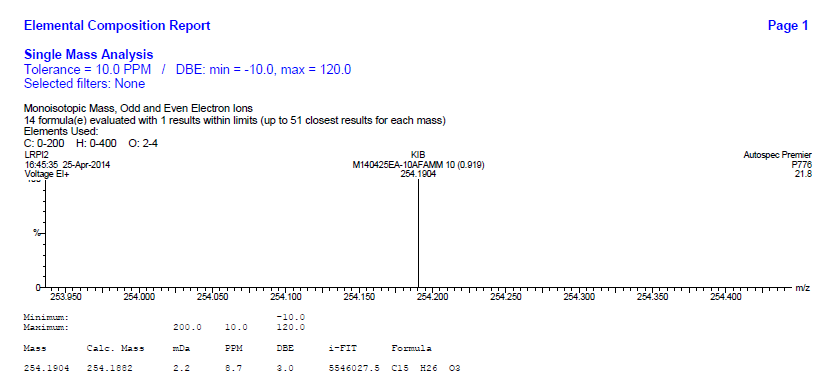


Figure 29S. ^1^H NMR (600 MHz, methanol-*d_4_*) spectrum of compound **5**


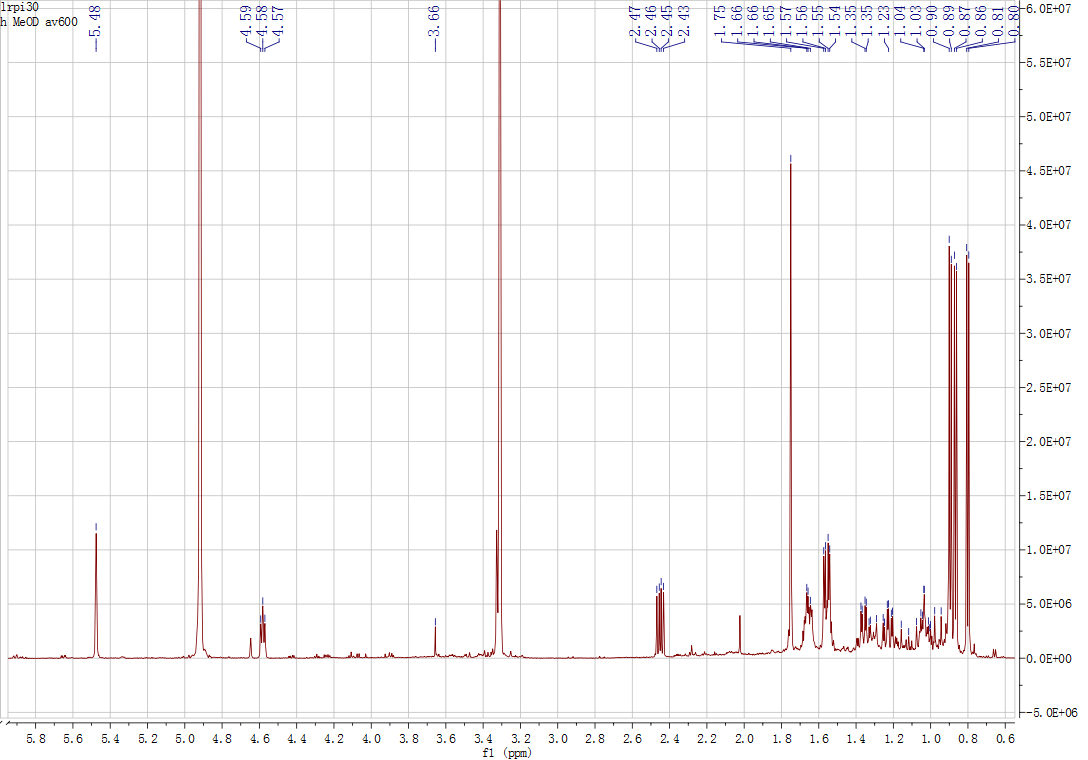


Figure 30S. ^13^C NMR (150 MHz, methanol-*d_4_*) spectrum of compound **5**


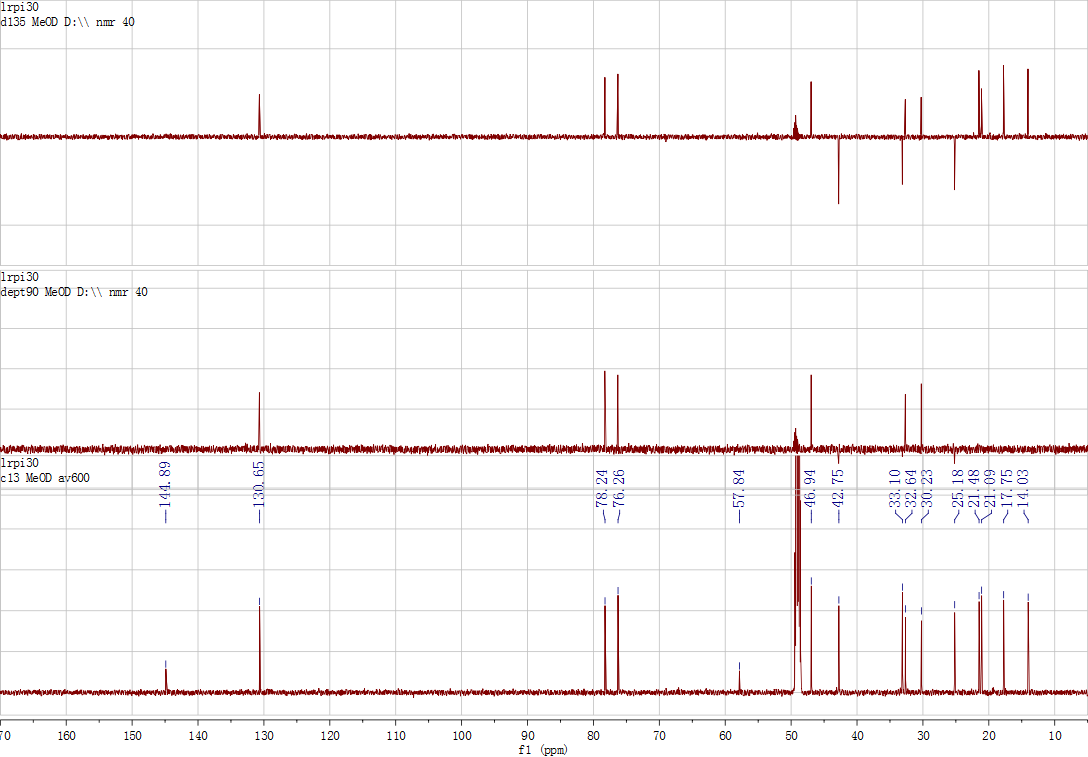


Figure 31S. ^1^H-^1^H COSY spectrum of compound **5**


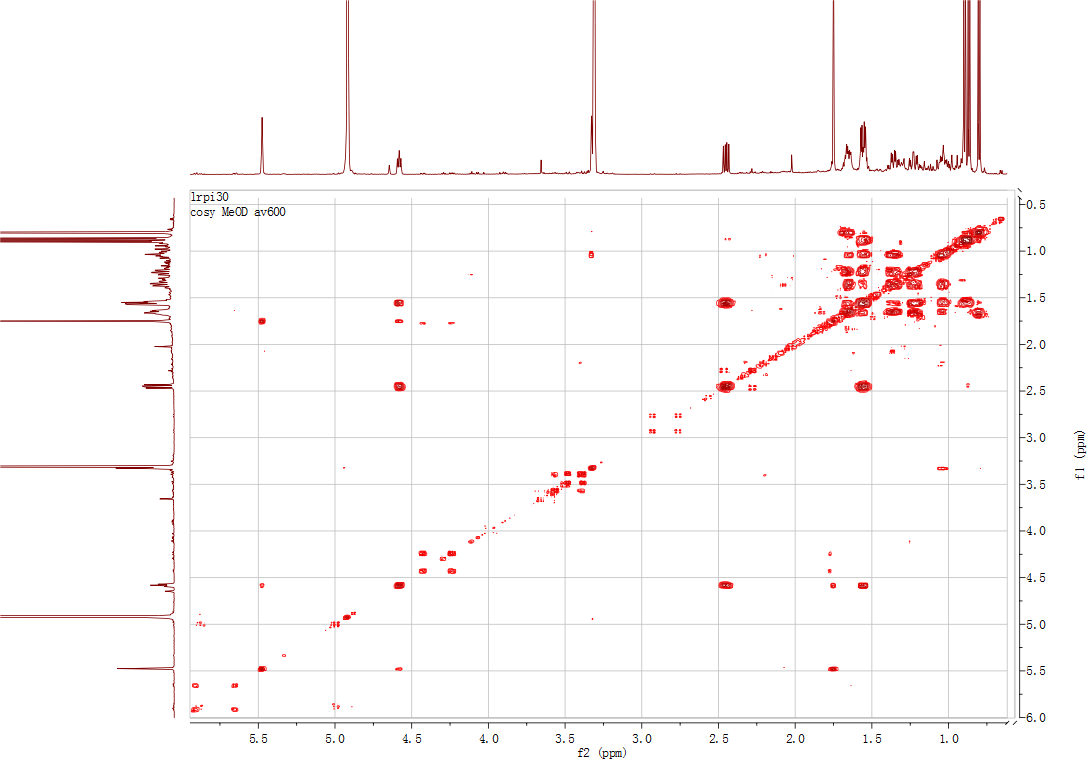


Figure 32S. HQSC spectrum of compound **5**


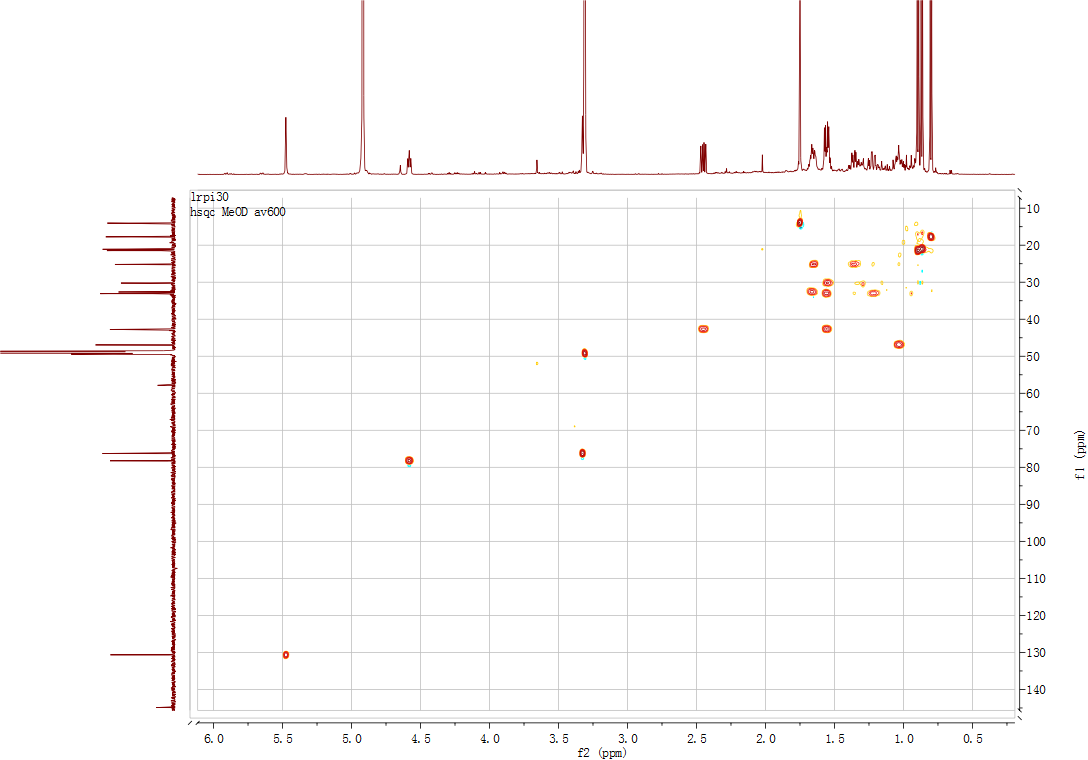


Figure 33S. HMBC spectrum of compound **5**


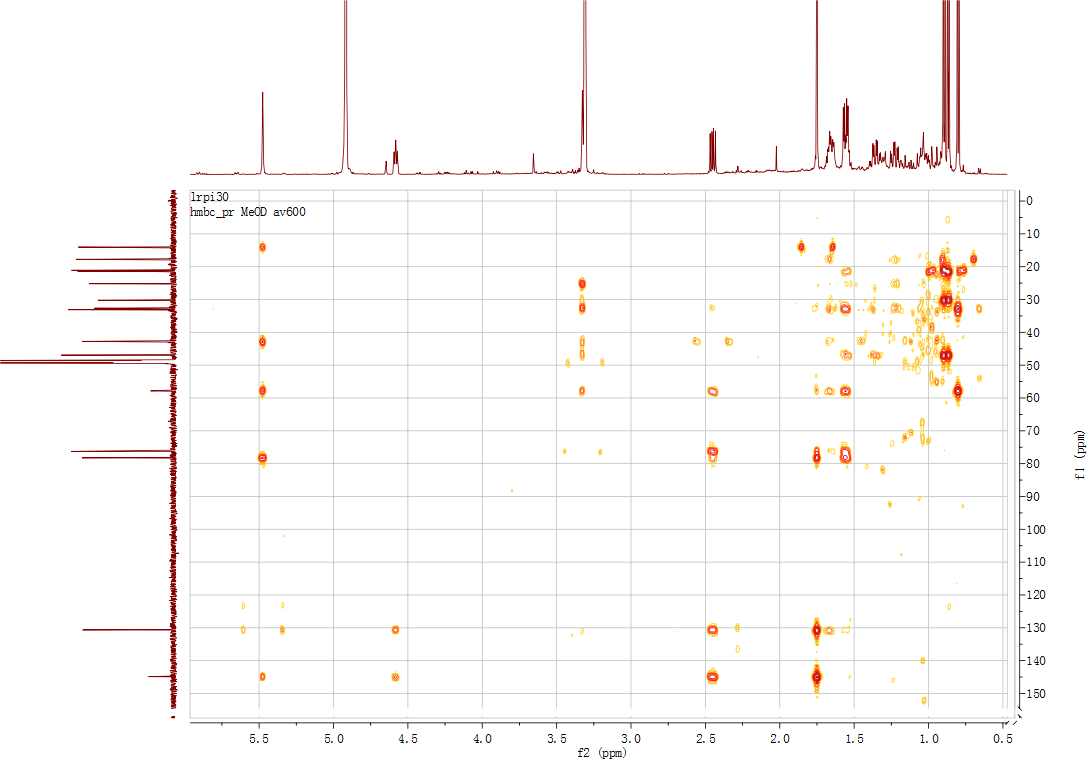


Figure 34S. ROESY spectrum of compound **5**


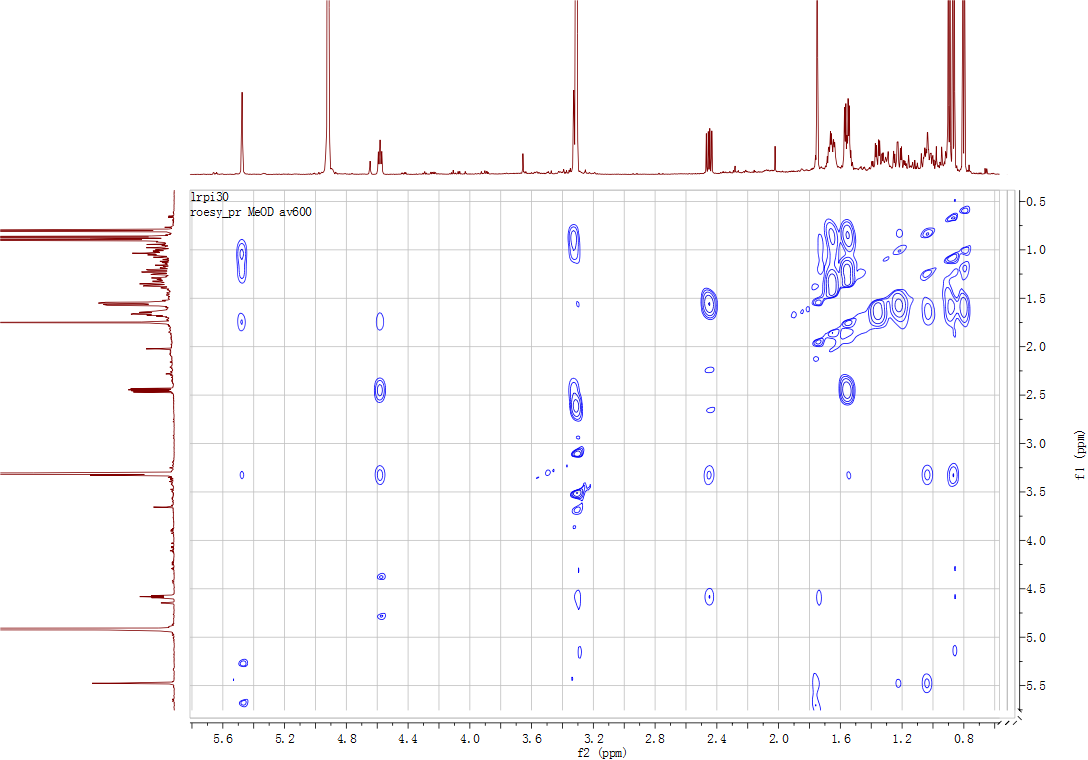


Figure 35S. HRESIMS of compound **5**


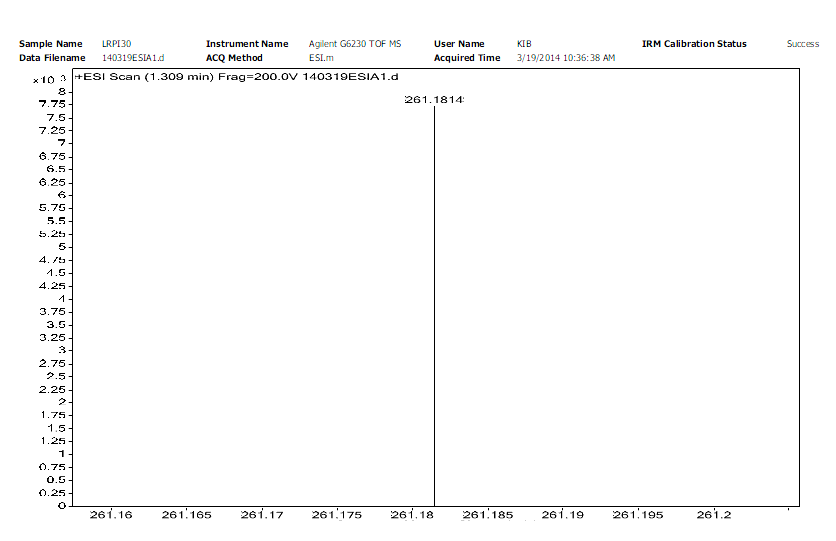


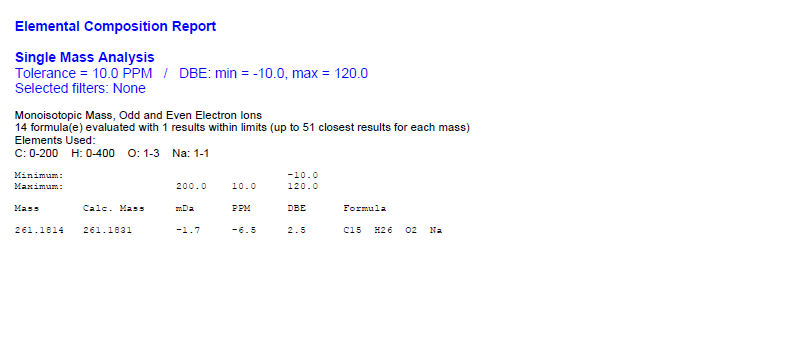

Supplement: Supplementary file 1 — Supplementary material 1 (DOCX 2983 kb) [file 13659_2014_45_MOESM1_ESM.docx]
